# Photocatalytic Transfer Hydrogenation Using Plastic Hydrolysates as Hydrogen Donor

**DOI:** 10.1002/anie.4324362

**Published:** 2026-05-04

**Authors:** Papa K. Kwarteng, Afreen H. Naceruddin, Erwin Reisner

**Affiliations:** ^1^ Yusuf Hamied Department of Chemistry University of Cambridge Cambridge UK

**Keywords:** acid hydrolysis, photocatalysis, plastic depolymerization, plastic upcycling, transfer hydrogenation, waste donors

## Abstract

The synthesis of aromatic amines requires harsh conditions or the use of fossil‐derived hydrogen (H_2_). Here, we address this limitation by demonstrating photocatalytic transfer hydrogenation (PTH) of nitroarenes into anilines employing plastic hydrolysates as electron and proton (hydrogen) donors under ambient temperature and pressure. PTH is achieved using a cobalt‐promoted molybdenum sulfide (^Co^MoS_2_) electrocatalyst integrated with a carbon nitride (CN_x_) semiconductor photocatalyst in acidic aqueous solution. ^Co^MoS_2_ reduces nitroarenes to anilines at –0.7 V versus RHE with a Faradaic yield of 70% and superior activity to platinum. The ^Co^MoS_2_‐CN_x_ photocatalyst produces anilines under simulated solar light (AM 1.5 G, 25°C), achieving 83%–99% yield from 24 nitroarenes using 4‐methylbenzyl alcohol as a model hydrogen donor. Acid hydrolysis of condensation polymers provides a source of alcoholic monomers in aqueous solution that can be used as a sustainable hydrogen donor for PTH in >80% yield using AM 1.5G or LED (405 nm, 33 mW cm^−2^) irradiation. A technoeconomic analysis (TEA) at pilot scale producing 1 t aniline day^−^^1^ using polyethylene terephthalate (PET) reveals a cut in cradle‐to‐gate emissions by ∼77% using PTH with ^Co^MoS_2_‐CN_x_ compared to conventional Pd/C hydrogenation with H_2_ from steam methane reforming (SMR‐H_2_) and a revenue‐generating levelized cost of aniline (LCOA) when co‐produced with terephthalic, acetic, and formic acids.

## Graphical abstract

Plastic waste is transformed into functional amines via solar‐driven transfer hydrogenation. Soluble monomers from acid hydrolysis of waste polymers serve as a hydrogen (electron/proton)donors in the selective reduction of nitroarenes using a visible light active photocatalyst consisting of cobalt promoted molybdenum disulfide integrated in cyanamide functionalized carbon nitride (^Co^MoS_2_‐CN_x_) under mild, ambient conditions.

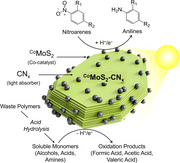

## 1 Introduction

The catalytic hydrogenation of nitroarenes to anilines offers a direct route to valuable nitrogen‐containing building blocks [1, 2]. Anilines serve as precursors in the manufacture of dyes, polymers, active pharmaceutical ingredients, agrochemicals, and advanced functional materials, making their sustainable synthesis a priority for the chemical industry [3]. Conventional hydrogenation methods predominantly rely on molecular hydrogen (H_2_) in combination with heterogenous noble metal catalysts such as platinum, palladium, or ruthenium, often requiring elevated temperatures and pressures to ensure high activity and selectivity [4]. Current advances in hydrogenation chemistry focus on replacing noble metals with less costly and more scalable alternatives such as Co, Ni, and Mo using H_2_ at high pressures [5]. These energy‐intensive conditions paired with the use of H_2_ produced from fossil fuels present intrinsic safety and environmental concerns as well as economic and energy security risks in decentralized or resource‐limited settings [6].

Transfer hydrogenation has emerged as a compelling alternative to those limitations, offering safer and more practical conditions by replacing molecular H_2_ with hydrogen donors such as alcohols, carboxylic acids, hydrazine, or sodium borohydride [7, 8, 9, 10, 11, 12]. Unlike direct hydrogenation with H_2_, transfer hydrogenation reactions proceed under ambient conditions and often allow for better functional group tolerance and catalyst recyclability [13, 14]. However, these methods still rely on non‐renewable, or hazardous reactants and the use of precious metal catalysts such as ruthenium or palladium [15, 16, 17]. A sustainable strategy to nitroarene reduction needs to replace fossil‐derived reagents and precious metal catalysts to ensure chemical circularity.

Photocatalysis has emerged as a powerful strategy to use solar energy to drive hydrogenation reactions [18, 19]. Both, molecular and semiconductor photocatalysts have been employed to demonstrate hydrogenation chemistry via single‐electron transfer [20, 21], or proton‐coupled electron transfer mechanisms [22], offering light‐driven access to protons and low potential electrons (H^+^/e^–^) [23]. Recent developments in PTH have demonstrated the viability of using alcohols and formic acid as hydrogen donors under visible light [9, 12, 24]. However, most of the reported PTH systems rely on precious metals and sacrificial electron donors that are either derived from petrochemical sources or unsuitable for large‐scale deployment due to their high cost, non‐scalability, volatility, toxicity, or waste generation [9]. PTH remains largely constrained to nitroarene to aniline reductions using well‐defined small‐molecule donors [14], with waste streams such as plastics not being previously explored as an electron or proton source for PTH.

Photoreforming (PR) exploits the oxidative valorization of organic waste substrates, but it has primarily been studied in the context of chemical fuel production, namely H_2_ generation [25, 26, 27], and, more recently, CO_2_ reduction [28, 29]. Most systems are designed to maximize the H_2_ yield from aqueous alcohols, biomass, or plastic hydrolysates using semiconductors such as TiO_2_ or carbon nitrides [30, 31]. The use of PR in promoting selective organic reductions remains largely unexplored, including its use in transfer hydrogenation. The use of plastic waste‐derived hydrolysates as reductant for photocatalytic organic reductions has also not yet been realized.

In recent work [25], we demonstrated that acid hydrolysis is an industrially relevant strategy for depolymerizing waste plastics such as textiles into photo‐reformable products, with the added benefit of acid recoverability and high H_2_ evolution activity. We showed excellent photocatalyst stability and performance of ^Co^MoS_2_ as an acid stable hydrogen evolution reaction (HER) co‐catalyst on the light absorbing CN_x_ semiconductor for condensation polymer waste upcycling under acidic conditions. Our findings also demonstrated that the acidic conditions enhanced photocatalyst activity, leading to higher H_2_ yields compared to alkaline or pH neutral conditions using the precious metal free photocatalyst, reaching an apparent quantum efficiency of 9%. Importantly, in pursuit of sustainability and circularity, we employed sulfuric acid from spent lead‐acid batteries as a sustainable and industrially relevant hydrolysis catalyst, further reinforcing the environmental viability of the process [25].

**SCHEME 1:**
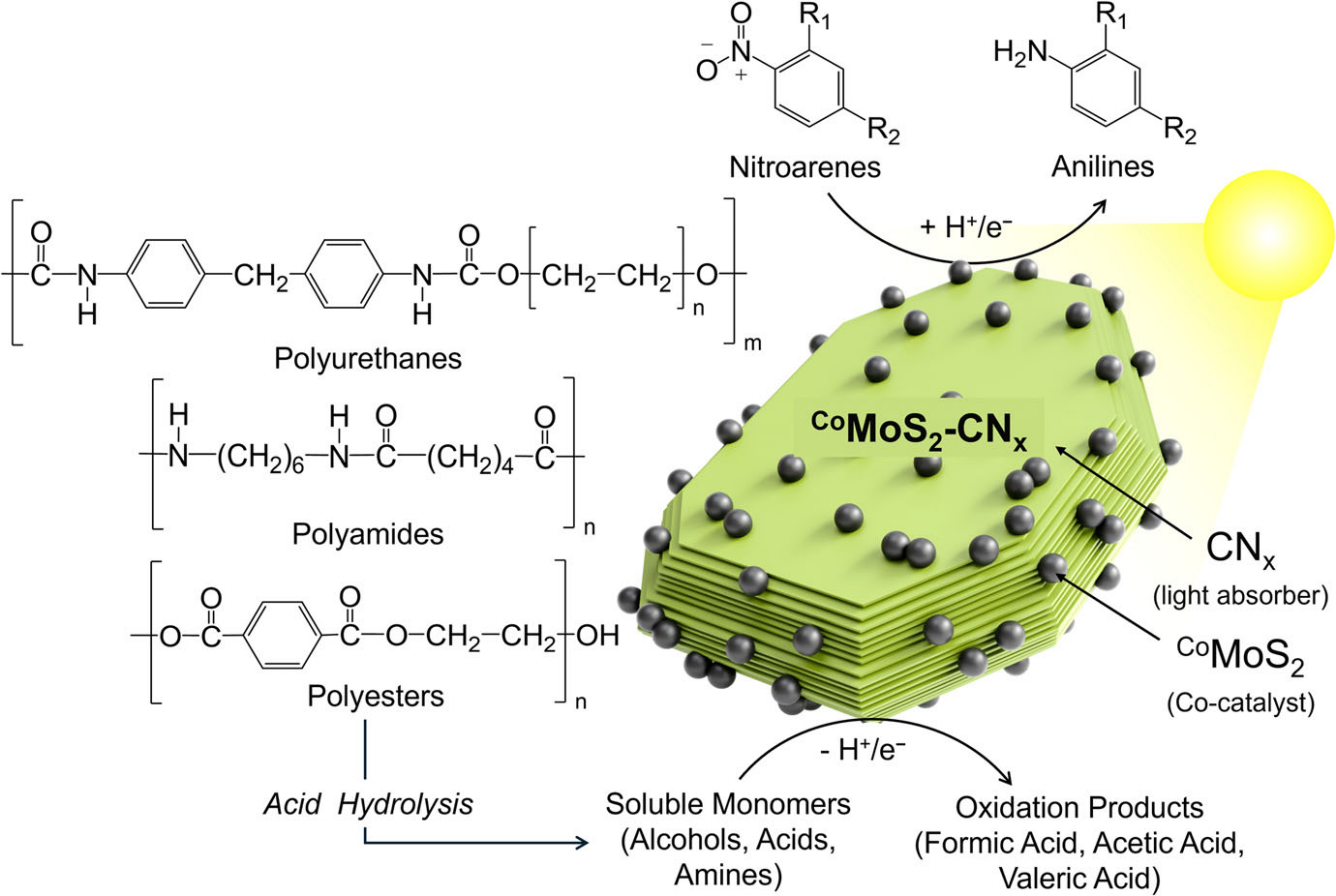
Schematic representation of photocatalytic transfer hydrogenation (PTH), showing the generation of alcohols and amines from acid hydrolysis of synthetic polymers including polyesters, polyamides and polyurethanes and their subsequent utilization as hydrogen donors for the PTH of nitroarenes to anilines. The photocatalysis is powered by sunlight and enabled by ^Co^MoS_2_, an in situ integrated co‐catalyst, within a cyanamide functionalized carbon nitride photocatalyst (CN_x_).

Here, we report the use of acid‐depolymerized polyester (e.g., polyethylene terephthalate, PET), polyamide (e.g., nylon 66), and polyurethane (PU) hydrolysates as hydrogen (H^+^/e^–^) donors for PTH (Scheme 1). These plastics release small molecule electron donors upon acid hydrolysis that are utilized to drive PTH [25, 32, 33, 34, 35]. We first show that ^Co^MoS_2_ is a selective hydrogenation electrocatalyst in aqueous acidic solution and compare its performance to platinum. Integration within CN_x_ allows the catalyst (^Co^MoS_2_‐CN_x_) to be driven by light for selective transfer hydrogenation of nitroarenes to anilines. CN_x_ serves as the light absorber, generating h^+^/e^–^ upon light irradiation, whereas ^Co^MoS_2_ serves as the co‐catalyst that facilitates nitroarene binding and reduction. PTH with ^Co^MoS_2_‐CN_x_ does not rely on H_2_ gas, unsustainable electron donors or precious metals, but sources H^+^/e^–^ from hydrolysates of acid‐pretreated synthetic polymers under room temperature, pressure, and simulated solar irradiation. We present a technoeconomic and carbon footprint assessment that confirms that our method provides a hydrogenation strategy with commercial potential and reduced environmental impact.

## 2 Results and Discussion

### 2.1 Synthesis of ^Co^MoS_2_ Electrocatalyst

^Co^MoS_2_ was synthesized using our previously reported thiocyanate‐melt mediated route, where cobalt(II, III)oxide and molybdenum(VI)oxide (1:2 weight ratio) are thermally treated with potassium thiocyanate (KSCN) at 400–500°C under Ar [25]. The resulting catalyst was dispersed in dimethylformamide and drop‐cast as an ink onto graphite foil to give the electrocatalyst (see Methods).

Compositional analysis by inductively coupled plasma optical emission spectroscopy (ICP‐OES), supported by X‐ray diffraction (XRD), X‐ray photoelectron spectroscopy (XPS) and electron microscopy (Figures S1–S3, Table S1), supports a ternary Co‐Mo‐S phase containing 8.8 wt% Co, 28.0 wt% Mo, and 36.0 wt% S (other components include K, C, H, N, and O). These values correspond to a Co:Mo:S stoichiometry of approximately 0.5:1:4 (10 mol% Co, 19 mol% Mo, and 72 mol% S), giving an empirical structure of Co_0.5_MoS_4_. XRD patterns exhibit broad reflections characteristic of amorphous MoS_2_ and Co_x_S_y_‐like domains, pointing to a largely mixed metal sulfides structure containing possible MoS_x_ phases (Figure S1) [25]. The Mo 3d XPS region shows reduction of Mo^6+^ to Mo^4+^, consistent with the formation of MoS_2_‐type species, while the S 2p_3/2_ signal at ∼161 eV confirms the presence of S^2–^ in metal sulfide environments [36]. The Co 2p region contains mixed Co^2+^/Co^3+^ signals with their associated satellites, evidencing mixed cobalt sulfide domains within the material (Figure S2) [37]. TEM imaging reveals a highly dispersed, integrated Co‐Mo‐S network (Figure S3) also confirmed previously [25].

### 2.2 Electrochemical Characterization of ^Co^MoS_2_

Our motivation for evaluating nitrobenzene (NB) electroreduction using ^Co^MoS_2_ arises from recent reports showing that metal‐sulfide electrocatalysts can exhibit enhanced selectivity for transfer hydrogenation due to the strong and specific adsorption of nitro groups onto vacant sulfur sites [38]. Co‐species have also been reported as effective nitrate reduction electrocatalysts and their inclusion in ^Co^MoS_2_ could further promote efficient nitroarene reduction [39, 40]. A Co/Mo ratio of 0.5 provides a higher density of S‐vacancy clusters as reported in the literature [41], and given the amorphous nature of ^Co^MoS_2_, we hypothesize that this high sulfur‐vacancy density can sustain efficient and selective NB electroreduction even at high proton concentrations (low pH).

**FIGURE 1:**
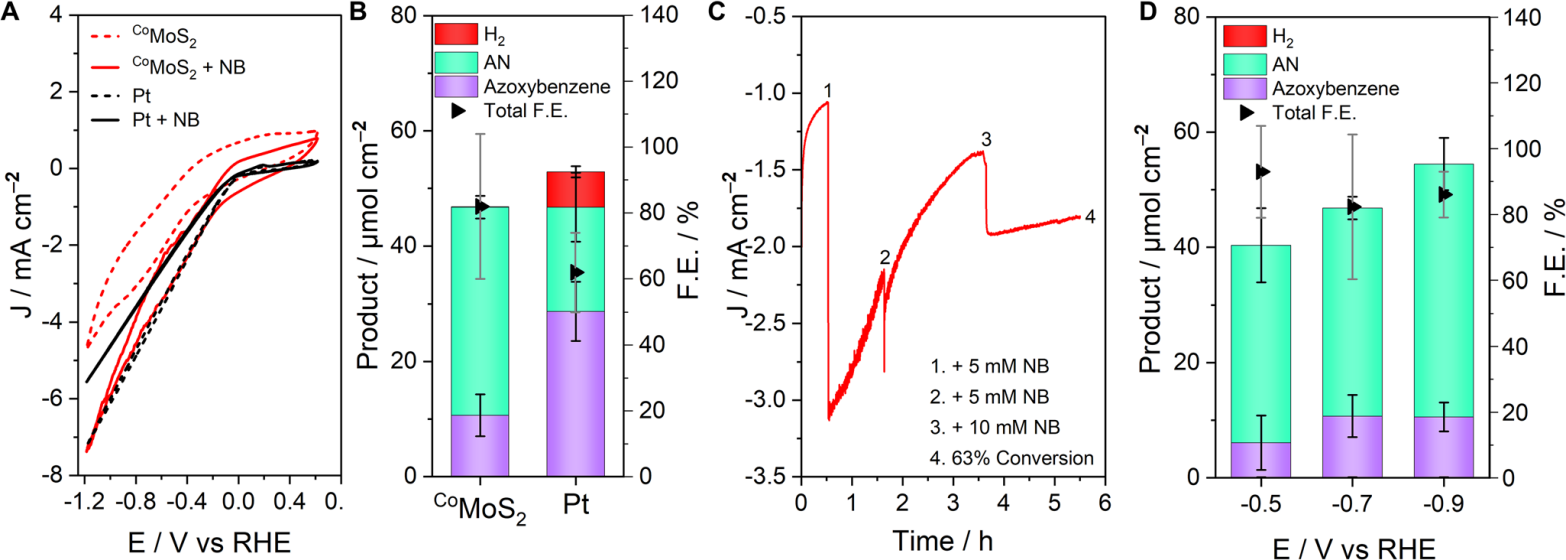
Electrocatalytic performance of ^Co^MoS_2_ for nitrobenzene (NB) reduction. (A) Cyclic voltammograms for ^Co^MoS_2_ and Pt in 0.1 M K_2_SO_4_ 1:1 H_2_O/MeCN (pH 2), with and without NB. ^Co^MoS_2_ shows an anodic shift in onset and a marked substrate‐enhanced catalytic wave in the presence of NB, whereas for Pt, NB reduction results in lower current density than proton reduction (HER). (B) Product distributions on ^Co^MoS_2_ and Pt after chronoamperometry (CA) at –0.7 V versus RHE. HER is completely suppressed using ^Co^MoS_2_ with aniline (AN) as the major product, whereas Pt produces more H_2_ and azoxybenzene. Total F.E. for ^Co^MoS_2_ was 82 ± 22% and 62 ± 12% for Pt. (C) Chronoamperometry with stepwise NB additions (5 and 10 mM) at −0.7 vs RHE give instantaneous, concentration‐dependent current steps, confirming a substrate‐limited catalytic current. (D) Potential dependent NB reduction on ^Co^MoS_2_ (‐0.5, ‐0.7, ‐0.9 V vs RHE) shows a window where AN selectivity is maximized over other NB reduction products. Total F.E. for ^Co^MoS_2_ at −0.5, −0.7, and −0.9 V versus RHE was 93 ± 14%, 82 ± 22%, and 86 ± 7%, respectively. Electrodes used are ^Co^MoS_2_ ink drop‐cast on 1 cm^2^ graphite foil; electrolyte, and procedures as in methods. The error bars were obtained from three independent experiments. The data are presented as mean values ± standard error of the mean.

A ^Co^MoS_2_ working electrode (1 cm^2^) was prepared by dispersing the synthesized ^Co^MoS_2_ powder in dimethylformamide followed by drop casting on graphite foil (see methods for more details). Cyclic voltammetry was performed with the ^Co^MoS_2_ working electrode using a three‐electrode configuration in a H_2_O:MeCN (1:1 v/v, 10 mL, 25°C) solvent mixture containing K_2_SO_4_ (0.1 M) and H_2_SO_4_ (0.01 M; see section on photocatalytic transfer hydrogenation for reason of solvent choice) with a Ag/AgCl (saturated NaCl) reference electrode and a Pt mesh counter electrode. The ^Co^MoS_2_ working electrode showed good HER background activity and the addition of NB (20 mM) results in an anodic onset shift and a further increase in cathodic current from –4.7 to –7.4 mA cm^−2^ at –1.2 V versus RHE, evidencing a substrate‐responsive catalytic wave (Figure 1A). In comparison, the electrochemical behavior of a Pt mesh working electrode displays the opposite response, showing a good current density from HER in the absence of substrate with a decrease in current response upon addition of NB (from –7.2 to –5.6 mA cm^−2^).

Product analysis after chronoamperometry (CA) experiments using quantitative ^1^H NMR spectroscopy for liquid‐phase products and gas chromatography (GC) for H_2_ confirms the preferential reduction of NB on ^Co^MoS_2_, whereas proton reduction dominates on Pt. After 3 h of CA at –0.7 V versus RHE in the presence of NB, ^Co^MoS_2_ produced 36 ± 2 µmol cm^−2^ aniline (AN) with a Faradaic efficiency (FE) of 70%, along with azoxybenzene (11 ± 4 µmol cm^−2^, 12% FE) and negligible H_2_ (Figures 1B, S4, and Tables S2,S3). The selectivity of NB reduction over HER is likely due to the enhanced adsorption of the nitro‐group in NB on sulfur vacancies that exist within ^Co^MoS_2_ [38]. In contrast, Pt generated 6 ± 1 µmol cm^−2^ of H_2_ (3% FE), 29 ± 5 µmol cm^−2^ of azoxybenzene (30% FE), and 18 ± 6 µmol cm^−2^ of AN (29% FE), consistent with high H^*^ coverage blocking nitro/intermediates adsorption and affecting selectivity [42]. Other identified side products included azobenzene and reductive intermediates like phenylhydroxylamines (not quantified due to overlapping ^1^H NMR peaks).

Pt is an established benchmark electrocatalyst for hydrogenation under H_2_ atmospheres, but remains less effective as a transfer hydrogenation electrocatalyst, likely due to its non‐selective reduction and preferential HER activity under electrochemical conditions [43, 44, 45]. To further demonstrate the preferential reduction of NB over protons on a ^Co^MoS_2_ cathode, we performed stepwise NB additions at –0.7 V versus RHE with 5 mM added after 0.5 h; another 5 mM after 1.0 h and another 10 mM after 2.0 h. The addition of NB results in instantaneous and concentration‐dependent current increases, followed by gradual decay as NB is consumed on the ^Co^MoS_2_ working electrode (Figure 1C).

Potential‐dependent CA on ^Co^MoS_2_ (Figures 1D, S5, and Tables S4–S5) shows an increase in overall conversion yield when moving to more cathodic potentials, while maintaining a good selectivity for AN from strong specific NB adsorption [38]. Changing the potential from –0.5 to –0.9 V versus RHE increases the AN yield from 34 ± 6 µmol cm^−2^ (85% FE) to 44 ± 5 µmol cm^−2^ (74% FE), with a concomitant increase in azoxybenzene yield from 6 ± 5 µmol cm^−2^ (8% FE) to 10 ± 3 µmol cm^−2^ (12% FE). HER remains suppressed relative to organic reduction on ^Co^MoS_2_ with a FE <1% at all potentials.

Electrochemical NB reduction to AN under acidic conditions has rarely been reported, with most works utilizing polyoxometalate redox mediators for the reduction of the nitro‐group in phosphoric acid electrolytes [46, 47]. The ability of ^Co^MoS_2_ to sustain selective reduction of nitroarenes to anilines in acidic aqueous solution without the need of an additional reagent highlights its promise as a non‐noble metal electrocatalyst for sustainable transfer hydrogenation. Thus, we establish ^Co^MoS_2_ as a rare and efficient nitroarene transfer hydrogenation electrocatalyst under acidic conditions with excellent chemoselectivity toward AN. We therefore selected ^Co^MoS_2_ as a co‐catalyst for cyanamide functionalized carbon nitride (CN_x_) in the PTH experiments.

### 2.3 Photocatalytic Transfer Hydrogenation

We synthesized the photocatalyst ^Co^MoS_2_‐CN_x_ by thermal polymerization of cobalt and molybdenum oxides with melamine (CN_x_ precursor) at 500°C, followed by KSCN salt–melt treatment at 400°C to introduce the cyanamide functionality and enable in situ sulfidation of the metal oxides as previously reported (see Methods for synthesis details) [25].

To establish and optimize PTH reaction conditions, we first use 4‐methylbenzyl alcohol (4MBA) as a model electron donor due to its established clean two‐electron oxidation to 4‐methylbenzaldehyde (4MBAd) to enable electron accounting with the six‐electron reduction of NB to AN, resulting in the production of 3 mol equivalents of 4MBAd per mol of AN [22, 23, 25]. Upon light irradiation (hν ≥ ∼2.7 eV) [25], ^Co^MoS_2_‐CN_x_ generates conduction band electrons and valence band holes; the holes oxidize 4MBA to 4MBAd, releasing electrons and protons and suppressing charge recombination, while the electrons reduce NB to AN [48, 49].

Using NB (20 mM) and 4MBA (100 mM) under AM 1.5G irradiation at 25°C, we first screened the MeCN/H_2_O (pH 2) solvent ratio to balance photocatalyst (^Co^MoS_2_‐CN_x_, 20 mg, 1 mL) dispersibility, proton availability and substrate solubility (Figure S6, Table S6) [50]. For PTH, the electron donor 4MBA is added in excess of the nitro substrate as typical in transfer hydrogenation reactions of nitroarenes [7, 12]. After 24 h, using quantitative ^1^H NMR spectroscopy analysis, NB conversion was >90% when using 5% to 70% H_2_O (pH 2) in MeCN. The AN yield varied due to transient nitroso/phenylhydroxylamine build‐up that can limit complete reduction [51, 52].

**FIGURE 2:**
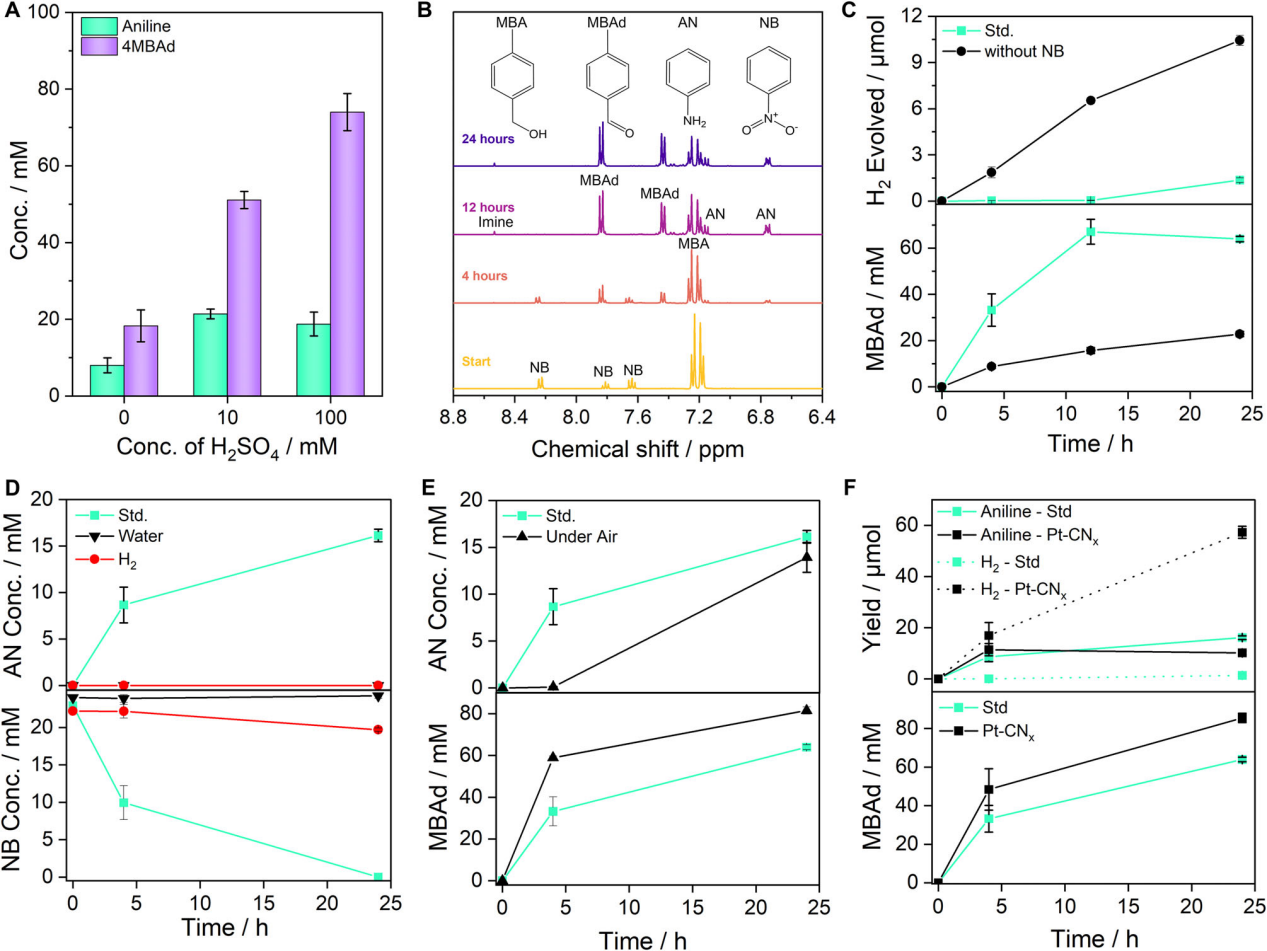
Photocatalytic transfer hydrogenation (PTH) of nitrobenzene (NB). (A) Effect of sulfuric acid (H_2_SO_4_) concentration on PTH activity. 4‐methylbenzyl alcohol (4MBA, 100 mM, 1 mL) is used as an electron donor with 20 mM NB as substrate and ^Co^MoS_2_‐CN_x_ as photocatalyst (20 mg). (B) ^1^H NMR tracking of reaction products from PTH of NB over time, showing yields at 4, 12, and 24 h with peaks of NB, aniline (AN), 4MBA, 4 methylbenzaldehyde (4MBAd) and a Schiff base (imine, Scheme S1) formed between 4MBAd and AN (^1^H NMR conditions are given in methods). (C ‐ F) Comparison of different reaction conditions with the standard conditions (Std., 100 mM 4MBA, 20 mM NB in 1:1 H_2_O:MeCN under N_2_ atmosphere). (C) H_2_ evolution and 4MBAd yield of ^Co^MoS_2_‐CN_x_ PTH catalyst with and without NB showing higher oxidation and reduction product yields in the presence of NB and a preferential transfer of electrons to NB over protons. (D) Comparison of PTH activity using different hydrogen donors: Std (4MBA), water, and molecular H_2_. Results show 4MBA as an effective donor for PTH. (E) PTH under N_2_ and under air reveals an initial lag in AN formation under air (sealed vessel) due to O_2_ scavenging by the photocatalyst, but shows comparable longer time yields to AN under N_2_. (F) Pt‐CN_x_ shows less selectivity and yield towards AN and produces more H_2_ than ^Co^MoS_2_‐CN_x_. The error bars were obtained from three independent experiments. The data are presented as mean values ± standard error of the mean.

We next examined the effect of pH on PTH because ^Co^MoS_2_‐CN_x_ exhibits enhanced activity at low pH [25], which makes it compatible with the acid‐catalyzed hydrolysis of condensation polymers. After 24 h and at neutral pH, the 4MBA was moderately oxidized, giving 18 ± 4 mM of 4MBAd and 8 ± 2 mM of AN with a total of ∼40% NB conversion. At pH 2 (0.01 M H_2_SO_4_), NB conversion exceeded 99%, with 21 ± 1 mM of AN and 51 ± 2 mM of 4MBAd produced. Thus, the acidic conditions used for plastic depolymerization are well compatible with PTH using ^Co^MoS_2_‐CN_x_. At pH 1 (0.1 M H_2_SO_4_), 19 ± 3 mM of AN (largely as anilinium), with 74 ± 5 mM of 4MBAd is produced (∼75% NB conversion, Figure 2A, Table S7). Acid‐enhanced 4MBA oxidation effectively couples the oxidation and reduction half‐reactions. We employed a 1:1 H_2_O:MeCN mixture containing aqueous H_2_SO_4_ (∼pH 2, 0.01 M) as our standard PTH conditions henceforth.

After establishing the standard conditions (Std.) as 1 mL solution containing 20 mM NB and 100 mM 4MBA (1:1 H_2_O:MeCN, pH 2) under AM 1.5G irradiation at 25°C under N_2_ atmosphere, we conducted time‐resolved experiments to track AN formation (Figure 2B, Table S8). Reaction progress was monitored using quantitative ^1^H NMR spectroscopy for liquid‐phase products and gas chromatography (GC) for H_2_ quantification. After 4 h, approximately 10 ± 2 mM of NB remained, giving 9 ± 2 mM of AN with a yield of 44% together with presumably partially reduced intermediates, nitrosobenzene and phenylhydroxylamine (detected in small amounts and not quantified) [23]. This is corroborated by the concurrent formation of 33 ± 7 mM of 4MBAd. After 12 h, the NB had almost disappeared (0.4 ± 0.1 mM), with the generation of 17 mM of AN (∼87% yield) and a minor imine side product (4 mM), resulting from Schiff base formation following condensation between 4MBAd and AN (Scheme S1) [49].

To evaluate the kinetics and selectivity of nitroarene reduction over proton reduction, we compared H_2_ evolution under standard conditions. In the presence of NB, no H_2_ was detected during the first 12 h of irradiation while NB was being consumed (AN yield of ∼87%, 17 mM). Once NB had been consumed, H_2_ was evolved (1.4 ± 0.2 µmol H_2_ after 24 h, Figure 2C, Table S9) consistent with the selectivity of the ^Co^MoS_2_ electrocatalyst observed from the electrochemical study. In the absence of NB, H_2_ evolved steadily from 1.9 ± 0.4 µmol H_2_ within 4 h to 10.4 ± 0.3 µmol H_2_ after 24 h. This observation confirms the preference of photocatalytic NB reduction over proton reduction on ^Co^MoS_2_‐CN_x_ in our solvent system as observed from the electrochemical study (Figure 1). The preferential reduction of NB was further confirmed using oxidation product yields: after 24 h, 23 ± 0 mM 4MBAd was produced in the absence of NB, whereas 64 ± 1 mM of 4MBAd was generated when NB was present.

No significant PTH of NB was observed under similar conditions (1:1 H_2_O:MeCN, pH 2) when water [53], or H_2_ was used as the electron donor. This control experiment confirms that electrons utilized in PTH are sourced from 4MBA (Figure 2D, Table S10). Operating the PTH under air (closed vessel) instead of nitrogen showed a 50% increase in 4MBA oxidation activity after 4 h with negligible AN formation, suggesting that atmospheric oxygen (O_2_) acted as an efficient electron scavenger until its consumption, which is then followed by NB reduction to 14 ± 2 mM of AN after 24 h (Figure 2E, Table S11). This result highlights the robustness of PTH under air in a sealed reaction vessel, eliminating the need for rigorous O_2_ abating steps.

Finally, we benchmarked ^Co^MoS_2_‐CN_x_ PTH activity with a Pt‐modified CN_x_ (1 wt% of Pt) photocatalyst prepared by a reported chemical reduction procedure using chloroplatinic acid as Pt precursor (see methods for details) [54]. Pt nanoparticles were well dispersed in the CN_x_ matrix as confirmed by TEM (Figure S7). ^Co^MoS_2_‐CN_x_ showed higher selectivity for AN from PTH of NB, maintaining over 95% selectivity compared to ∼40% AN selectivity from Pt‐CN_x_. Negligible H_2_ was detected using ^Co^MoS_2_‐CN_x_ compared to 17 ± 5 µmol H_2_ with the Pt‐CN_x_ system after 4 h of irradiation (Figure 2F, Table S12). After 4 h, the AN:4MBAd ratio was ∼1:4 for the ^Co^MoS_2_‐CN_x_ system befitting NB reduction steps, whereas the ratio was ∼1:5 for the Pt‐CN_x_ system indicating contributions from HER. After 24 h, the less selective Pt‐CN_x_ produced less AN overall (10 ± 1 mM) compared to ^Co^MoS_2_‐CN_x_ (16 ± 1 mM) and consistent with our electrochemical characterization (Figure 1). This comparison positions ^Co^MoS_2_‐CN_x_ as a selective and unique noble metal free PTH catalyst.

To further investigate the dependency of PTH performance on photocatalytic oxidation of donor substrates, we compared the performance of 4MBA as a donor to other conventional sacrificial and waste‐derived donors for NB (20 mM) reduction (Figure S8, Table S13, 100 mM of donor in 1 mL of solvent). Among the tested donors, triethanolamine (TEOA) [55] delivered the highest AN yield (∼100%), followed by glucose (76 ± 13%), glycerol (47 ± 1%) and ethylene glycol (EG, 36 ± 4%). The observed trend reflects the relative ease of photocatalytic oxidation of each donor.

### 2.4 Substrate Scope for PTH

**SCHEME 2:**
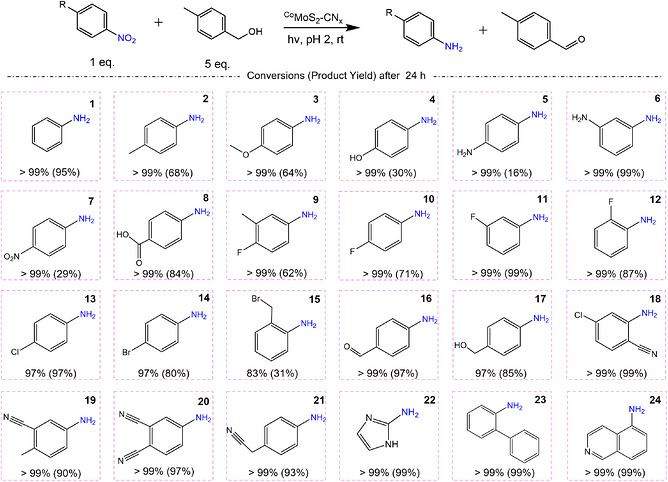
Substrate scope for the photocatalytic transfer hydrogenation (PTH) of nitroarenes. Conversion efficiencies and product yields (in brackets) are shown for nitroarene substrates subjected to AM 1.5 G irradiation for 24 h. Reaction mixtures contained nitroarene substrate (20 mM), 4MBA (100 mM), and H_2_SO_4_ (0.01 M, pH 2) in a H_2_O:MeCN (1:1 v/v) solvent mixture. 4MBA acts as electron donor for PTH. Conversion and product yields were quantified by ^1^H NMR spectroscopy.

After establishing the optimized reaction conditions and benchmarking the standard conditions of PTH using ^Co^MoS_2_‐CN_x_ with 4MBA and NB as electron donor and acceptor, respectively, we investigated the scope and functional group tolerance of the photocatalytic system. We selected a representative set of functional groups such as nitriles and aldehydes as electron‐withdrawing groups (EWGs), alcohols as electron‐donating groups (EDGs) and halogens (F, Cl, Br) bearing both EWG and EDG characteristics (Scheme 2) [56]. We used our standard conditions with nitroarene (20 mM) added to 1 mL of 4MBA (100 mM) and irradiation with AM 1.5G (25°C). After 24 h of irradiation, complete conversion was achieved for nearly all substrates (Scheme 2). Conversion was quantified based on the amount of substrate consumed using ^1^H NMR spectroscopy, with corresponding product yields shown in parentheses.

High yields were obtained for NB (**1**) and substrates with EDGs such as methyl, anisole, and amine moieties (**2**,**3**,**6**) as well as EWGs such as carboxylic acids, halogens, aldehydes and nitriles (**8–16**, **18–21**) underscoring the robustness of the system across diverse aromatic frameworks. Notably, halogenated nitroarenes (**9–15**, and **18**) were selected to probe the chemoselectivity of the ^Co^MoS_2_‐CN_x_ photocatalyst in the presence of reducible C–X bonds. In all cases, exclusive nitro group reduction was observed with full retention of the halogen substituents and no detectable dehalogenation or side reactions.

This high degree of chemoselectivity was further evident in multifunctional substrates **16–22**, where selective reduction of the nitro group was achieved even in the presence of aldehydes, alkenes, or nitriles. This selectivity stems likely from the preferential adsorption of nitro groups at vacant sulfur sites in ^Co^MoS_2_ and the ring activation toward nitro group reduction conferred by the strong electron withdrawing effect of the nitro group. Interestingly, substrate **17**, bearing both nitro and hydroxyl functionalities, afforded its corresponding amine without concurrent oxidation of the hydroxyl group, indicating excellent functional group tolerance. This is also likely due to the abundance of a more electron rich donor, 4MBA. Furthermore, the photocatalyst effectively reduced sterically hindered nitroarenes such as substrates **9**, **12**, and **18**, and maintained activity against bulky frameworks including extended aromatic systems (**23**) and fused rings (**24**). Imine formation from condensation of resulting anilines and 4MBAd [49] were observed in some substrates, but not investigated further (Scheme S1).

### 2.5 Synthetic Polymers as Hydrogen Donors

**FIGURE 3:**
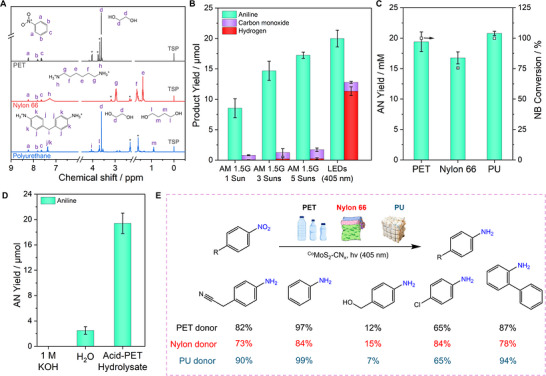
Photocatalytic transfer hydrogenation (PTH) of nitrobenzene (NB) enabled by plastic hydrolysates. (A) Representative ^1^H NMR spectra of polymer hydrolysates (8× diluted) containing NB (20 mM), highlighting soluble monomers released during depolymerization: ethylene glycol (EG) from PET, hexamethylenediamine from nylon 66, and EG and 1,4‐butanediol from polyurethane (PU, Figures S10–S12). * represents protons from unidentified products. (B) Yields of aniline (AN) obtained from PTH of NB using diluted PET hydrolysate (∼173 mM EG) as the hydrogen donor under different light sources and intensity to demonstrate versatility of the PTH process. Monochromatic 405 nm (33 mW cm^−2^) LEDs deliver the highest yields within 24 h. (C) PTH of NB to AN using acid hydrolysate of condensation polymers as hydrogen donors. (D) Comparison of AN yield using mimics of alkaline hydrolysate (1 M KOH, ∼200 mM EG), neutral hydrolysate (Water, ∼200 mM EG) and PET hydrolysate (∼173 mM EG, ~0.9 M H_2_SO_4_). Solvent used was 1:1 H_2_O:MeCN under 405 nm irradiation for 24 h at 25°C. (E) Mini scope of nitroarene substrates undergoing PTH using plastics under 405 nm LED irradiation. Reaction mixtures contained 20 mM nitroarene and diluted hydrolysate; conversion efficiencies were quantified by ^1^H NMR after 24 h irradiation. The error bars were obtained from three independent reactions. The data are presented as mean values ± standard error of the mean.

After demonstrating PTH of nitroarenes using 4MBA as a model electron donor, we next explored the possibility of using plastic waste streams as sustainable and scalable chemical reductants. Representative post‐consumer plastics, specifically PET (300 µm powder, semicrystalline >40% crystallinity), nylon 66 granules (Poly *N,N*’‐hexamethylene adipinediamide, hexamethylene adipamide) and PU elastomer (Elastollan 35A) were hydrolyzed in acid to generate water‐soluble monomers. 10 g of each was treated in 50 mL of H_2_SO_4_ (7.5 M) under reflux for 6 h. After hydrolysis, we obtained 1.4 M EG from PET, 2 M hexamethylenediamine, 0.3 M adipic acid from nylon 66 (bulk of adipic acid precipitates out of hydrolysate), and 0.35 M butanediol, 0.41 M EG, and 0.16 M methylenedianiline from PU (determined by quantitative ^1^H NMR spectroscopy, Figure 3A), consistent with literature [25]. Insoluble monomers such as terephthalic acid (TPA) from PET precipitated out cleanly at low pH underscoring another benefit of acid hydrolysis as a depolymerization strategy [25].

We used a standard PTH system using NB (20 mM) and PET hydrolysate as the hydrogen (H^+^/e^–^) donor (diluted 8× with 1:1 H_2_O:MeCN, ~0.9 M H_2_SO_4_) to demonstrate versatile operation under sunlight irradiation (simulated) and indoor artificial lighting (LEDs). 20 mg of ^Co^MoS_2_‐CN_x_ was used with 1 mL of diluted plastic hydrolysate and irradiated for 24 h in all cases. Under simulated solar light AM 1.5 G (~100 mW cm^−^^2^) illumination, the catalyst produced an AN yield of 43% (9 ± 2 mM) with negligible H_2_ evolution. Compared to PTH using aqueous 4MBA, ^Co^MoS_2_‐CN_x_ yielded 2.5× less AN when EG‐derived from PET was used as a donor indicating a strong dependence on  the donor type utilized in PTH. However, increasing the solar intensity to 3 and 5 suns using a Fresnel lens increased the AN yield to 73% (15 ± 2 mM) and 86% (17 ± 1 mM), respectively, demonstrating that PTH proceeds efficiently under concentrated simulated sunlight. Notably, monochromatic 405 nm LED (33 mW cm^−2^) irradiation, near the absorption maximum of ^Co^MoS_2_‐CN_x_ further increased activity, delivering a 97% AN yield along with higher H_2_ evolution. This shows that PTH is compatible with both solar and monochromatic LED excitation, and that matching the excitation wavelength to the photocatalyst's optical absorption leads to enhanced charge‐carrier utilization and higher product yields (Figure 3B, Table S14). 405 nm irradiation produced higher yields because the wavelength lies within the maximum absorption region of ^Co^MoS_2_‐CN_x_ (Figure S9).

Under monochromatic irradiation, hydrolysates from PET, nylon 66 and PU produced 19 ± 2, 17 ± 1, and 21.0 ± 0.3 mM of AN, respectively, corresponding to 97%, 76%, and 99% AN yield (Figures 3C, S10–S12, and Table S15). We propose that the soluble monomers after acidic polymer hydrolysis undergo photocatalytic oxidation to release electrons and protons. For example, from the PET hydrolysate, we expect a two‐electron oxidation of EG to glycolaldehyde as the main oxidation pathway, followed by subsequent oxidation, dehydration, rearrangement and decarbonylation steps that can yield formic acid, acetic acid and carbon monoxide (CO) [25]. Supporting ^1^H NMR data confirmed the presence of formic acid, implicating glycolaldehyde as potential precursor to CO (Figures S13–S16, Table S16) [57, 58]. Using labelled ^13^C EG, we confirmed the presence of ^13^CO after photocatalysis under the same conditions (Figure S17). Electrons released from plastic monomer oxidation drive NB reduction to nitrosobenzene followed by a second reduction step still powered by monomer oxidation to *N*‐phenylhydroxylamine and finally a third two‐electron step then reduces the hydroxylamine to AN, completing the six‐electron transfer cascade [20]. While the low potential electrons are immediately utilized for NB reduction and protons from the acidic aqueous solution are used for transfer hydrogenation, protons released from alcohol oxidation can in principle become the proton source during long‐term PTH.

To highlight the importance of acid hydrolysis as a pretreatment strategy prior to photoreforming, we compared PTH under acidic conditions (97% yield, 19 ± 2 mM AN) with PTH under alkaline and neutral conditions at the optimized conditions (containing ∼200 mM EG). The comparison was performed under 405 nm irradiation (24 h) using 20 mg of ^Co^MoS_2_‐CN_x_ and 1 mL of hydrolysate or EG solutions under alkaline or neutral conditions. Under alkaline conditions (1 M KOH), simulating conventional alkaline hydrolysates, no AN was detected due to phase separation between aqueous EG and acetonitrile which led to NB remaining in the organic MeCN phase and ^Co^MoS_2_‐CN_x_ in the aqueous phase (Figures 3D, S18, and Table S17). Only 3 ± 1 mM of AN was obtained under pH neutral conditions, a fraction of what was achieved using the acid hydrolysate, emphasizing the dual advantage of acid hydrolysis.

To probe the system's chemoselectivity using obtained hydrolysates, we performed PTH on a range of nitroarene substrates under monochromatic 405 nm irradiation (25°C, 24 h). Product yields for AN from NB were higher with PET (97%) and PU hydrolysates (99%) than nylon 66 hydrolysates (84%), possibly because of their rich concentration of alcohols which serve as effective donors for PTH. All hydrolysates performed poorly for the selective PTH of nitrobenzyl alcohol as the corresponding aminobenzyl alcohol is a more favorable aromatic donor substrate than the plastic monomers (Figure 3E) [59]. Crucially, no product formation was observed in the absence of light, catalyst or hydrolysate solution, underscoring the necessity of each component for PTH (Table S18).

### 2.6 Economic and Environmental Analysis

**FIGURE 4:**
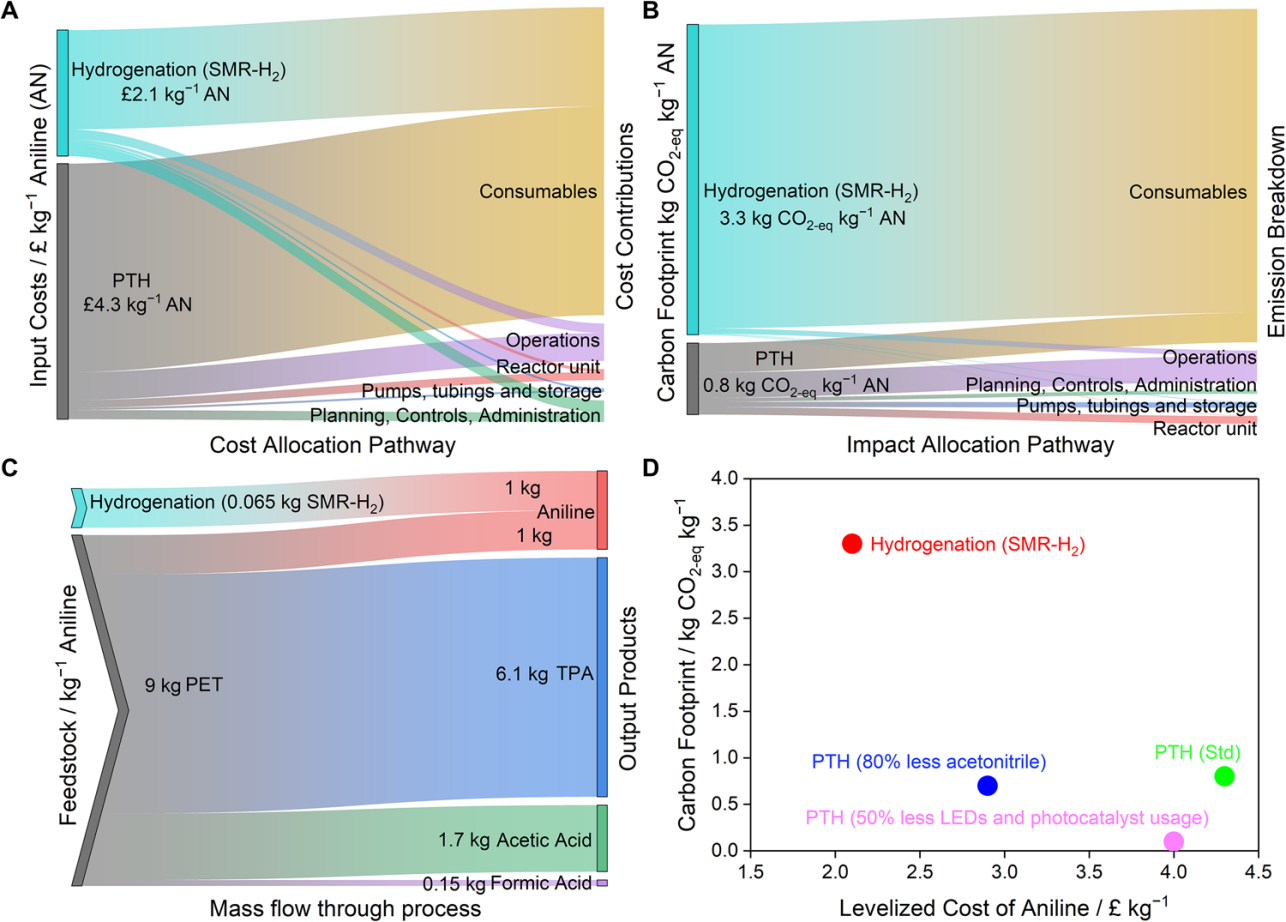
Technoeconomic and environmental comparison of conventional and photocatalytic transfer hydrogenation (PTH) routes to aniline (AN). (A) Cost and (B) cradle‐to‐gate carbon footprint contributions for aniline (AN) production (1 t AN day^−^^1^) by process category over a 20‐year plant life. (C) Material flow Sankey showing input output balance and co‐product generation in the PTH system. (D) Cost–carbon frontier comparing industrial hydrogenation and PTH configurations. Figure shows PTH is not only environmentally advantageous, but also approaches economic viability under realistic process configurations.

To evaluate the feasibility of PTH beyond laboratory scale toward competing with current hydrogenation technologies, we performed a combined technoeconomic and environmental assessment at a pilot‐scale throughput of 1‐ton AN day^−^^1^ (see supplementary discussion for details, Figure 4, Tables S19–S31). The process flow comparison in Figure S19 illustrates the fundamental distinction between the two routes: While industrial Pd/C hydrogenation requires fossil‐derived H_2_ from steam methane reforming (SMR), high‐pressure compression and external H_2_ recycling, PTH integrates PET hydrolysis with light‐driven transfer hydrogenation so that EG generated during depolymerization acts as the hydrogen donor for NB.

Cost breakdowns considering AN as a product but disregarding hydrolysis and oxidation products from PET (Figure 4A) indicate that the levelized cost of AN (LCOA) for PTH is currently higher than that of Pd/C hydrogenation (£4.3 vs £2.1 kg^−^^1^ AN) mainly constituted by consumables cost. In contrast, costs in the Pd/C route are dominated by the price of SMR‐derived H_2_. Environmental analysis reveals that cradle‐to‐gate emissions fall by ∼77% relative to the Pd/C–SMR baseline (0.8 vs 3.3 kg CO_2‐eq_ kg^−^^1^ AN) when using PTH (Figure 4B). These reductions derive primarily from displacing fossil H_2_ production and incorporating carbon avoided for removing PET from waste streams and incineration. Despite the higher cost of AN with PTH, mass‐flow analysis shows that PET upcycling simultaneously generates multiple value‐added co‐products, including TPA from PET hydrolysis, formic and acetic acids from PET PR alongside the targeted AN (Figure 4C). When these co‐product revenues are incorporated, the effective LCOA for PTH becomes negative (Table S26), transforming the system into a net revenue‐generating platform.

Finally, scenario analysis (considering only AN as product) demonstrates that improvements such as using 80% less acetonitrile and reducing LED and photocatalyst costs by 50% shift PTH into a region of both lower carbon intensity and competitive LCOA relative to the Pd/C benchmark (Figure 4D, Table S31). When co‐products of PTH (TPA, formic and acetic acids) are factored in, AN cost is neglible indicating an increased revenue stream from PTH (Table S26). These projections place PTH on a new cost–carbon frontier, where waste mitigation, commodity chemical production, and greenhouse‐gas reduction are all aligned within a single manufacturing platform. Together, the analyses in Figure 4 show that PTH is not only environmentally advantageous, but also potentially economically viable under simulated process configurations and has the potential to complement or displace conventional aromatic amine hydrogenation within emerging circular‐economy and carbon‐pricing frameworks.

## 3 Conclusions

This work introduces a sustainable platform for plastic waste utilization through organic transfer hydrogenation via acid photoreforming. Rather than using sacrificial electron donors and chemical reductants like previous photocatalytic hydrogenation reports, we employ hydrolysates of post‐consumer polymers as hydrogen (H^+^/e^−^) donors and achieve selective photocatalytic transfer hydrogenation of nitroarenes under ambient pressure and temperature without precious metal catalysts, pressurized hydrogen or elevated temperature. The acid‐stable integrated photocatalyst ^Co^MoS_2_‐CN_x_ manages precise electron and proton transfer to the unsaturated organic substrate, achieving efficient hydrogenation of the nitro‐moiety with excellent yields and functional group tolerance using either simulated solar light (AM 1.5 G) or monochromatic LEDs (405 nm) at ambient temperature and pressure. The successful integration of ^Co^MoS_2_ into other light absorbers such as TiO_2_, WO_3_, and SrTiO_3_ could provide useful integrated photocatalysts for various applications under low pH conditions in future development. Beyond our experimental demonstration, we support our study with an economic and environmental feasibility study that integrates plastic waste photoreforming with catalytic hydrogenation chemistry. The technoeconomic analysis shows that by scaling to industrially relevant throughputs, the plastic‐derived photocatalytic system can approach cost parity with conventional hydrogenation while delivering substantial reductions in carbon intensity (∼77%). Together, the tandem acid plastic hydrolysis–photocatalytic transfer hydrogenation process establishes a novel chemical process scenario for circular chemical manufacturing.

## Conflicts of Interest

A patent application (GB 2606571.4, 25^th^ March 2026) covering this work has been filed by Cambridge Enterprise that names P.K.K. and E.R. as inventors.

## Supporting information



The authors have included supporting information which includes methods, supplementary discussion, Tables S1–S31 and Figures S1–S19.
**Supporting File**: anie72081‐sup‐0001‐SuppMat.pdf.

## Data Availability

The data that support the findings of this study are available from the University of Cambridge data repository: https://doi.org/10.17863/CAM.128814.
