# Supplementary material for: Photocatalytic Transfer Hydrogenation Using Plastic Hydrolysates as Hydrogen Donor
Source: Angew Chem Int Ed Engl. 2026 May 4;65(25):e4324362. doi: 10.1002/anie.4324362 (PMC13266921; doi:10.1002/anie.4324362)
Supplement: Supplementary file 1 — The authors have included supporting information which includes methods, supplementary discussion, Tables S1–S31 and Figures S1–S19. Supporting File: anie72081‐sup‐0001‐SuppMat.pdf. [file ANIE-65-e4324362-s001.pdf]

Supporting Information

Photocatalytic Transfer Hydrogenation Using Plastic Hydrolysates as Hydrogen Donor

Papa K. Kwarteng[a], Afreen H. Naceruddin[a], Erwin Reisner[a],*

[a] Mr. Papa K. Kwarteng, Ms. Afreen H. Naceruddin, Prof. Erwin Reisner
Yusuf Hamied Department of Chemistry
University of Cambridge
Lensfield Road, Cambridge CB2 1EW, UK
E-mail: reisner@ch.cam.ac.uk

* Corresponding author

Supplementary Discussion

Technoeconomic comparison of aniline production routes

Photocatalytic transfer hydrogenation (PTH) using PET-derived ethylene glycol

Photocatalytic transfer hydrogenation (PTH) offers a sustainable route for reductive organic transformations by replacing molecular hydrogen (H_2) with *in situ* generated H_2 equivalents from renewable donors such as waste plastics under light irradiation.[1-5] In this work, we demonstrate that coupling photoreforming of polyethylene terephthalate (PET) with the reduction of nitrobenzene (NB) to aniline (AN), achieves simultaneous polymer upcycling and fine chemical synthesis within a single integrated process. Ethylene glycol (EG) derived from the acid depolymerization of PET acts as the H_2 donor, while terephthalic acid (TPA) is recovered as a high purity co-product, ensuring minimal waste generation and closing the carbon loop.[4]

From technoeconomic modelling, we consider a pilot plant producing 1-ton AN day^{-1} from 9-ton PET feedstock, with a 20-year operational lifetime (Tables S19-S31). PET depolymerization (7.5M H_2SO_4, 140 °C) achieves 90% efficiency, generating 2 tons of EG and 6.1 tons TPA per day, with the acid reused up to four cycles before replacement. The hydrolysate is diluted 4× with water and mixed 1:1 with acetonitrile, giving requirements of 33,750 L H_2O daily and 45,000 L MeCN recycled yearly with 10% makeup, circulated through 9,000 m^2 of flat panel photoreactors (1 cm optical depth) containing 900 kg $^{Co}MoS_2$-CN_x photocatalyst (5 wt%, 10 mg mL^{-1}). Irradiation is provided by 9,000 solar-powered 405 nm LED floodlights, with uniform photon flux maintained across the array. Before reaction, the photoreforming solution is purged with N_2 to remove dissolved O_2, ensuring selective NB reduction:

$$C_6H_5NO_2 + 3C_4H_6O_2 \longrightarrow C_6H_5NH_2 + 3CH_3COOH + 2H_2O \quad (1)$$

The system operates continuously under ambient conditions, avoiding the high pressures (20-50 bar) and temperatures (200-300 °C) of conventional hydrogenation.[7-8] This eliminates compression energy losses and simplifies reactor safety design while enabling modular scalability. The energy demand arises mainly from hydrolysis, LED operation, and catalyst synthesis. Based on experimental and modelled inputs, the net process carbon intensity is estimated to be 0.8 kg $CO_{2\text{-eq}}$ kg inherently reduces compression losses and reactor safety requirements while allowing modular scaling. The system's energy input arises mainly from hydrolysis, LED operation, and photocatalyst synthesis.[4] The net process carbon intensity is estimated at 0.8 kg $CO_{2\text{-eq}}$ kg^{-1} aniline, corresponding to a 77% reduction relative to conventional Pd/C hydrogenation (3.3 kg $CO_{2\text{-eq}}$ kg^{-1} aniline). The levelized cost of aniline

(LCOA) is £4.3 kg^{-1}, dominated by solvent makeup, LED costs, and catalyst replacement. However, when co-product revenues from TPA (£0.9 kg^{-1}), acetic acid (£1.3 kg^{-1}) and formic acid (£0.5 kg^{-1}) are included, the net LCOA becomes negative (- £0.4 kg^{-1}), indicating a profitable, low carbon process. This inversion underscores the multi-output advantages of PTH, where polymer hydrolysates provide both electrons and marketable co-products.

Overall, PTH demonstrates the feasibility of coupling waste plastic valorization with fine-chemical synthesis under sunlight. While challenges remain in large-area reactor integration and solvent recovery, the system establishes a blueprint for distributed, low carbon manufacturing.

Industrial hydrogenation of nitrobenzene to aniline

Industrial AN production is almost exclusively produced by the catalytic hydrogenation of NB using molecular H_2.[8] The overall reaction proceeds according to:

$$C_6H_5NO_2+3H_2 \longrightarrow C_6H_5NH_2+2H_2O \quad (2)$$

Commercial processes operate in either liquid or vapor phase configurations.[9] In the liquid-phase route, H_2 is dissolved in NB under moderate pressures (6 – 30 bar) and temperatures of 150 – 240 °C in the presence of supported metal catalysts, most commonly palladium, or platinum on carbon or alumina.[9] The vapor-phase process employs higher temperatures (250 – 300 °C) with gaseous feeds over fixed-bed catalysts.[10] Both configurations achieve > 99% conversion and > 90% selectivity towards aniline under optimized conditions. The reaction effluent is cooled, unreacted H_2 is separated and recycled, and water generated during hydrogenation is decanted. Crude AN is subsequently purified by fractional distillation to >99.9 wt% purity.

At a steady-state operation, the stoichiometric consumption of H_2 is three molar equivalents per mol of AN, corresponding to 0.07 kg H_2 per kg of AN produced. For a production rate of 1-ton AN per day, this equates to approximately 1323 kg of NB and 65 kg H_2 per day. The reaction is strongly exothermic, and the generated heat is typically recovered to preheat the feed, contributing to overall energy efficiency.[9] Auxiliary steam and cooling water are nonetheless required for start-up, temperature control and product separation.

H_2 for industrial hydrogenation is predominantly supplied via steam methane reforming (SMR), which remains the most cost-effective route.[11] The international Energy Agency's Global Hydrogen Review 2023 reports that grey hydrogen from SMR carries a carbon intensity of 10 – 12 kg CO_2 kg^{-1} H_2.[12]

For technoeconomic modelling, we analyze a Pd/C system operating at 60 bar at 50 °C in methanol. The ratio of Pd/C to NB and solvent was adapted from literature as 0.05 g Pd/C for

10 g of NB and 100 ml of methanol.[13] For 1 ton AN per day, this requires 6.6 kg of Pd/C (0.5 kg day^{-1}, assuming a reusability of 14 days),[5] and 13,230 L of methanol each day.[13] The levelized cost of AN (LCOA) depends primarily on H_2 feedstock prices, as well as utilities and fixed capital costs. The process' carbon footprint is dominated by H_2 production: SMR H_2 generation accounts for majority of total life-cycle emissions. As a result, any transition to low-carbon or renewable H_2 sources would directly reduce the environmental impact of AN manufacture. The industrial NB to AN route thus provides a well-defined, verifiable baseline for comparison with alternative photocatalytic systems.

Methods

Reagents

Melamine (99%), potassium thiocyanate (99%), sodium borohydride (98%), trisodium citrate dihydrate, molybdenum (VI) oxide (99.97%), cobalt (II,III) oxide (powder <10 μm), 4-methyl benzyl alcohol (98%), D-(+)-Glucose, formic acid, ethylene glycol (anhydrous, 99.8%), glycerol (>99.5%), triethanolamine, chloroplatinic acid solution (8 wt%), 4-nitroanisole, 4-nitrobenzoic acid, 1,4-dinitrobenzene, 2-nitroaniline, 4-nitroaniline, 4-nitrophenol, 1-fluoro-3-nitrobenzene, 2-fluoro-5-nitrotoluene, o-fluoronitrobenzene, 4-nitrophenylacetonitrile, 2-nitroimidazole, 2-nitrodiphenyl, 5-nitroisoquinoline, 1-chloro-4-nitrobenzene, 3-nitrobenzaldehyde, 4-chloro-2-nitrobenzonitrile and deuterium oxide (99.9 atom % D, contains 0.75 wt% 3-(trimethylsilyl)propionic-*2,2,3,3-d*$_4$ acid, sodium salt) were purchased from Sigma-Aldrich and used without further purification. Nitrobenzene, 1-fluoro-4-nitrobenzene, 2-methyl-5-nitrobenzonitrile and 1-bromo-4-nitrobenzene were purchased from fluorochem and used without further purification. 4-nitrotoluene and 4-nitrophthalonitrile were purchased from Alfa Aesar and used without further purification. 2-Nitrobenzyl bromide and 4-nitrobenzyl alcohol were purchased from Acros Organics and used without further purification.

Polymers

PET powder (300 μm, semicrystalline >40% crystallinity) were purchased from Goodfellow Cambridge Limited, Nylon 66 pellets (Poly N,N'-hexamethylene adipinediamide, Poly (hexamethylene adipamide) were obtained from Sigma Aldrich and Polyurethane thermoplastic elastomer (Elastollan 35A) was obtained from BASF.

Synthesis Methods

$^{Co}MoS_2$. 250 mg of cobalt (II,III) oxide was added to 500 mg of molybdenum (IV) oxide and 1.5 g of potassium thiocyanate. The mixture was ground together until even and heated to 400 °C under inert atmosphere at a ramp rate of 30 °C per min followed for 1 hour by a second heating to 500 °C at the same ramp rate for 30 mins. The obtained cake was crushed and ground into powders and washed thrice, twice with deionized water and once with 50:50 deionized water and ethanol mixture, centrifuged and dried overnight at 80 °C.

$^{Co}MoS_2$-CN_x. 83.3 mg cobalt (II, III) oxide was added to a mixture of 166.7 mg of molybdenum (IV) oxide and 4.75 g of melamine in a crucible and heated at 1 °C per minute until 500 °C for 3 hours. The resulting cake was ground into powders and denoted as ^{Co}Mo-gCN$_x$. 1.5 g of ^{Co}Mo-gCN$_x$ and 3 g of potassium thiocyanate were mixed and ground together until even and heated to 400 °C under inert atmosphere at a ramp rate of 30 °C per min followed for 1 hour by a second heating to 500 °C at the same ramp rate for 30 mins. The obtained cake was

crushed and ground into powders and washed thrice, twice with deionized water and once with 50:50 deionized water and ethanol mixture, centrifuged and dried overnight at 80 °C.

CN_x. Cyanamide functionalized carbon nitride was synthesized by weighing 5 g melamine into a crucible and heated at 1 °C per min to 500 °C for 3 hours. The obtained cake was crushed and ground into fine powders and denoted as gCN_x. 1.5 g of gCN_x and 3 g of KSCN were mixed and ground together until even. The solid mixture was placed in a boat crucible and heated to 400 °C under inert atmosphere at a ramp rate of 3 °C per min followed by a second heating to 500 °C at the same ramp. The obtained cake was crushed and ground into powders and washed thrice, twice with deionized water and once with 50:50 deionized water and ethanol mixture, centrifuged and dried overnight at 80 °C. The sample was denoted as CN_x.

Pt-CN_x. 150 mg of CN_x was dispersed in 10mL deionized water via sonication for 20 minutes. 0.29 g of trisodium citrate dihydrate ($C_6H_9Na_3O_7$ •$2H_2O$) was added to the dispersion and further sonicated for 20 minutes. 42 µL of hexachloroplatinic acid (H_2PtCl_6) was added to the dispersion and then further sonicated for 20 minutes and stirred for 20 minutes. 1 ml of 0.132 M sodium borohydride ($NaBH_4$) was added to the mixture and further stirred for 20 minutes before the powders were separated under centrifugation and dried overnight at 80 °C.

Fabrication of $^{Co}MoS_2$-CN_x electrode. 20 mg of $^{Co}MoS_2$ was dispersed in 2 mL DMF solution containing 5 wt% Nafion[117] and ultrasonicated for 30 minutes. 100 µL of the ink was drop cast onto graphite foil (1cm × 1cm active area) and dried under air over 2 days.

Electrochemical Measurements. All electrochemical measurements were carried out using a BioLogic VSP-300 Multichannel Potentiostat in a standard gas-tight two-compartment H cell separated by a Nafion proton exchange membrane, at room temperature. 10 mL of a 1:1 water-acetonitrile solution containing 0.1 M K_2SO_4 and 0.01 M H_2SO_4 (pH 2) was used as the electrolyte, with 20 mM nitrobenzene added to the cathodic chamber prior to electrochemical studies. $^{Co}MoS_2$ electrode or Pt mesh was used as the working electrode, with Pt mesh and an Ag/AgCl (saturated NaCl) electrode as the counter and reference electrode respectively. Both the catholyte and anolyte were purged with N_2, containing 2% CH_4 internal standard, for 15 minutes before running electrochemical tests. The CV scans were performed at 25 mV s^{-1} scan rate at room temperature under stirring. The current obtained was normalized with respect to the geometrical surface area of the working electrode. All the potential recorded with the Ag/AgCl (saturated NaCl) reference electrodes have been converted to the RHE scale using the following equation:

$$E_{(RHE)} = E_{(Ag/AgCl)} + 0.197\ V + 0.059\ V \times pH \quad (3)$$

where $E_{(Ag/AgCl)}$ is the observed potential using the Ag/AgCl (saturated NaCl) reference electrode.

Product Detection and Quantification. The gaseous product (H_2) at the cathode was detected and quantified using a Shimadzu GC-2010 Plus Gas Chromatograph with ultrapure Helium as the carrier gas, by manually injecting 50 µL of headspace gas from the H-cell using an air-tight syringe (Hamilton, GASTIGHT). The Faradaic efficiency of the products was determined using the following equation where Z is the number of electrons required for the respective product formation, n is the number of moles of product formed, F is the Faraday constant (96,485 C mol^{-1}), and Q is the total amount of charge passed during the same time interval.

$$\text{FE [product] (\%)} = \frac{ZnF}{Q} \times 100 \qquad (4)$$

The liquid products were analyzed and quantified by ^{1}H and ^{13}C NMR using a Bruker DPX 400 MHz Spectrometer after each experiment, with D_2O containing 0.75 wt% 3-(trimethylsilyl) propionic-2,2,3,3-d_4 acid, sodium salt as the internal standard for calculation of the yield.

Photocatalytic Transfer Hydrogenation. Photocatalytic transfer hydrogenation using $^{Co}MoS_2$-CN_x was done using a Newport Oriel 150 W 100 mW cm^{-2} solar light simulator (solar light was concentrated using a Fresnel lens for experiments where needed) equipped with an air mass 1.5 global filter. IR irradiation was removed using a water filter. Reactions were performed at 25 °C maintained using constant water circulation. For the pH dependent experiments, 20 mg of photocatalysts were dispersed in 1 ml of 20 mM nitrobenzene with 100 mM 4-methylbenzyl alcohol (4MBA) and the pH adjusted with known concentrations of in a 4.48 ml borosilicate vial using 50:50 H_2O:MeCN. All samples were stirred at 600 rpm during irradiation for 24 hours. For photocatalytic transfer hydrogenation using model electron donors, 4MBA was substituted for with formic acid, glycerol, glucose, ethylene glycol or triethanolamine. Photocatalytic substrates screening was conducted using 100 mM 4MBA, 0.01 M H_2SO_4 and 20 mM of the nitroarene. Any H_2 generated was monitored by sampling out the headspace gas via gastight syringe (50 µL aliquot; Hamilton) and analyzed by gas chromatography (Shimadzu GC-2010 Plus) with a discharge ionization detector. All samples with 4MBA were purged under nitrogen.

Hydrolysis of Condensation Polymer Waste. 50 ml of 7.5 M H_2SO_4 was used for the acid hydrolysis of 8 g PET powder at 140 °C under stirring for 24 hours. Acid hydrolysis was optimized from previous literature reports on terephthalic acid (TPA) recovery from waste PET materials. Same conditions were applied for hydrolysis of Nylon 66 and polyurethane.

Plastic enabled photocatalytic transfer hydrogenation. Acid hydrolysate obtained from acid pretreatment of PET powder was mixed with acetonitrile (50 v/v %). Prior to mixing with acetonitrile, the hydrolysate was diluted 4× and 20 mM of nitrobenzene or nitroarene substrate was added to the mixture. Photocatalytic reactions were done using a Newport Oriel 150 W 100 mW cm^{-2} solar light simulator equipped with an air mass 1.5 global filter. IR irradiation was removed using a water filter. Reactions were performed at 25 ° C maintained using constant water circulation. For photocatalysis with 405 nm LEDs (104 mW), 3 mL aliquots of solution mixture were delivered into crimp-cap vials sealed with septa and placed under N_2 containing 2 vol.% CH_4 as an internal standard for 10 minutes. The vials were then placed in the photoreactor (Treellum Technologies, Patent EP17382313) with a thermostatically controlled heating block at 25 °C with 250 rpm orbital rotation; wherein the vials were illuminated with 405 nm light from 104 mW LEDs. The reaction headspace was sampled via gastight syringe (50 µL aliquot; Hamilton) and analyzed by gas chromatography (Shimadzu GC-2010 Plus) with a discharge ionization detector. H_2 peak areas were converted to moles using calibrated response factors and 2 vol.% CH_4 in N_2 as internal standard.

Treatment of data. All analytical measurements were performed in triplicate, and are given as the unweighted mean ± standard deviation (σ). All measurements are listed as H_2 yield per weight of catalyst (µmol H_2 g^{-1}) and activity per weight of substrate (µmol g_{sub}^{-1} h^{-1}). σ was calculated via eq 3:

$$\sigma = \sqrt{\frac{\sum (x - \bar{x})^2}{n-1}} \qquad (5)$$

where n is the number of repeated measurements, x the value of a single measurement, and $\bar{x}$ the unweighted mean of the measurements.

Characterization

Elemental Analysis. Elemental analysis was performed using Inductively Coupled Plasma Optical Emission Spectroscopy (ICP-OES). A ThermoScientific iCAP 7400 ICP-OES DUO spectrometer was used to analyze the elemental composition such as sulfur as well as transition metals (Mo, Co) in the synthesized catalyst.

UV-Vis. UV-vis spectra were recorded on an Agilent Cary 60 UV-Vis spectrophotometer using a diffuse reflectance accessory. The measured diffuse reflectance of the mixture is then inverted directly by the program using the Kubelka–Munk theory.

Nuclear Magnetic Resonance (NMR) Spectroscopy. ^{1}H NMR spectra were collected on a 400 MHz Bruker spectrometer. Samples after photocatalytic HER and photoreforming of EG/

PET hydrolysate were used as is without further dilution. Hydrolysates from acid hydrolysis were diluted 10× before submitting for ^{1}H NMR analysis. Samples with 1 M acid or base concentration were put in thick-walled NMR tubes before submission.

XRD. Powder XRD measurements were performed by a Panalytical X'Pert Pro (K alpha Cu radiation) diffractometer using 1° min^{-1} scan rate.

XPS. XPS data was acquired on a Thermo Scientific Escalab 250Xi fitted with a monochromatic aluminum Kα X-ray source (1486.7 eV) at a pressure below 10^{-8} Torr and a room temperature of 294 K

Quantitative ^{1}H NMR Spectroscopy. Samples (typically 570 μL) were spiked with 30 μL of deuterium oxide (99.9 atom % D, containing 0.75 wt% 3-(trimethylsilyl) propionic-*2,2,3,3-d*$_4$ acid, sodium salt) as standard after hydrolysis, photocatalysis or photoreforming depending on the experiment. The concentration of analyte in the sample was calculated via eq. 1:

$$C_{Analyte}=\frac{I_{Analyte}}{I_{Standard}}\frac{N_{Standard}}{N_{Analyte}}C_{Standard}.F \qquad (6)$$

Where $C_{Analyte}$ is the concentration of the analyte peak, $C_{Standard}$ is the concentration of the standard peak, I_{analyte} the integral of the analyte peak area, $I_{Standard}$ the integral of the standard peak area, N_{analyte} the number of protons corresponding to the analyte peak, $N_{Standard}$ the number of protons corresponding to the standard peak and F is the ratio of total volume to volume of sample.

pH Measurements. pH was measured on a Mettler Toledo pH meter and probe. Samples in concentrated acid were diluted 10× prior to measuring to avoid damaging the probe, and the pH was back calculated by using the assumption that a[H^+] ≈ c[H_2SO_4]

Supplementary Tables

Table S1 | Compositional elemental analysis and inductively coupled plasma optical emission spectroscopy (ICP-OES) showing contents of carbon, hydrogen, nitrogen, cobalt, molybdenum and sulfur present in synthesized $^{Co}MoS_2$ electrocatalyst and $^{Co}MoS_2$-CN_x.

Element	Composition / % $^{Co}MoS_2$	Composition / % $^{Co}MoS_2$-CN_x (95% CN_x)
C	0.3	22.8
H	0.7	1.2
N	0.5	38.3
K	0.2	13.9
Co	8.8	1.2
Mo	28.0	5.1
S	36.0	3.4
O	25.5	14.1

Table S2 | Product analysis performed post chronoamperometric (CA) studies. Chronoamperometry was performed at –0.7 V vs RHE for 3 hours. Initial nitrobenzene concentration was 20 mM. H_2 was determined using a gas chromatograph with 2% methane as internal standard whereas liquid products were determined using quantitative ^{1}H NMR with 0.75 wt% trimethylsilylpropionic acid (TSP) in D_2O as internal standard. Electrolyte consisted of 0.01 M H_2SO_4, 0.1 M K_2SO_4 at room temperature under stirring. Electrode area is 1 cm^2

Electrode	Hydrogen Conc. / µmol	Aniline Conc. / µmol	Azoxybenzene Conc. / µmol
$^{Co}MoS_2$	0.09 ± 0.01	36 ± 2	11 ± 4
Pt Mesh	6 ± 1	18 ± 6	29 ± 5

Table S3 | Faradaic efficiencies of products post chronoamperometric (CA) studies. Chronoamperometry was performed at -0.7 V vs RHE for 3 hours. Initial nitrobenzene concentration was 20 mM. H_2 was determined using a gas chromatograph with 2% methane as internal standard whereas liquid products were determined using quantitative ^{1}H NMR with 0.75 wt% trimethylsilylpropionic acid (TSP) in D_2O as internal standard. Electrolyte consisted of 0.01 M H_2SO_4, 0.1 M K_2SO_4 at room temperature under stirring. Electrode area is 1 cm^2.

Electrode	Hydrogen	Aniline	Azoxybenzene	Total F.E. / %
$^{Co}MoS_2$	~ 0%	70 ± 22 %	12 ± 1 %	82 ± 22 %
Pt Mesh	3.2 ± 0.4 %	29 ± 10 %	30 ± 6 %	62 ± 12 %

Table S4 | Product analysis performed post chronoamperometric (CA) studies. Chronoamperometry was performed at different potentials for 3 hours using $^{Co}MoS_2$ electrode. Initial nitrobenzene concentration was 20 mM. H_2 was determined using a gas chromatograph with 2% methane as internal standard whereas liquid products were determined using quantitative ^{1}H NMR with 0.75 wt% trimethylsilylpropionic acid (TSP) in D_2O as internal standard. Electrolyte consisted of 0.01 M H_2SO_4, 0.1 M K_2SO_4 at room temperature under stirring. Electrode area is 1 cm^2

Potential / V vs RHE	Hydrogen Conc. / µmol	Aniline Conc. / µmol cm^{-2}	Azoxybenzene Conc. / µmol cm^{-2}
–0.5	0.08 ± 0.02	34 ± 6	6 ± 5
–0.7	0.09 ± 0.01	36 ± 2	11 ± 4
–0.9	0.08 ± 0.01	44 ± 5	10 ± 3

Table S5 | Faradaic efficiencies of products post chronoamperometric (CA) studies. Chronoamperometry was performed at different potentials for 3 hours using $^{Co}MoS_2$ electrode. Initial nitrobenzene concentration was 20 mM. H_2 was determined using a gas chromatograph with 2% methane as internal standard whereas liquid products were determined using quantitative ^{1}H NMR with 0.75 wt% trimethylsilylpropionic acid (TSP) in D_2O as internal standard. Electrolyte consisted of 0.01 M H_2SO_4, 0.1 M K_2SO_4 at room temperature under stirring. Electrode area is 1 cm^2

Potential / V vs RHE	Hydrogen	Aniline	Azoxybenzene	Total F.E.
- 0.5	~ 0%	85 ± 13 %	8 ± 4 %	93 ± 14 %
- 0.7	~ 0%	70 ± 22 %	12 ± 1 %	82 ± 22 %
-0.9	~ 0%	74 ± 6 %	12 ± 4 %	86 ± 7 %

Table S6 | Solvent ratio (H_2O:MeCN) effect on photocatalytic activity and yield of aniline from nitrobenzene hydrogenation. 4MBA (100 mM, 1 mL) is used as proton and electron donor with 20 mM nitrobenzene as substrate under AM 1.5G irradiation at room temperature and pressure for 24 hours. 20 mg of photocatalyst was used for each of the reactions and reaction solutions contained 0.01 M H_2SO_4. Products were determined using quantitative ^{1}H NMR with 0.75 wt% trimethylsilylpropionic acid (TSP) in D_2O as internal standard.

Water in acetonitrile / %	Nitrobenzene Conversion / %	4MBA Conversion / %	Aniline Yield / mM	4MBAd Yield / mM
70	99	48 ± 4	12 ± 4	41 ± 0
50	99	49 ± 2	21 ± 2	52 ± 4
30	99	48 ± 3	17 ± 1	42 ± 3
10	98 ± 2	47 ± 2	14 ± 0	39 ± 6
5	91 ± 9	50 ± 1	14 ± 2	40 ± 7

Table S7 | Effect of sulfuric acid concentration on photocatalytic activity and yield of aniline from nitrobenzene hydrogenation. 4MBA (100 mM, 1 mL) is used as proton and electron donor with 20 mM nitrobenzene as substrate. Experiments were performed under simulated AM 1.5G irradiation at room temperature and pressure for 24 hours using 20 mg of catalyst in 1:1 H_2O:MeCN as solvent under N_2. Products were determined using quantitative ^1H NMR with 0.75 wt% trimethylsilylpropionic acid (TSP) in D_2O as internal standard.

Sulfuric acid Conc. / M	4MBAd Conc. / mM	Aniline Conc. / mM
0	18 ± 4	8 ± 2
0.01	51 ± 2	21 ± 1
0.1	74 ± 5	19 ± 3

Table S8 | Liquid-phase products and reactant consumption from photocatalytic transfer hydrogenation over time using ^1H NMR. 4MBA (100 mM, 1 mL) is used as proton and electron donor with 20 mM nitrobenzene as substrate. Experiments were performed under simulated AM 1.5G irradiation at room temperature and pressure over a 24-hour period using 20 mg of $^{Co}MoS_2$-CN_x in 1:1 H_2O:MeCN under N_2. Products at each timepoint were determined using quantitative ^1H NMR with 0.75 wt% trimethylsilylpropionic acid (TSP) in D_2O as internal standard.

Time / h	Nitrobenzene Conc. / mM	4MBA Conc. / mM	Aniline Conc. / mM	4MBAd Conc. / mM	Imine Conc. / mM
0	23 ± 0	94 ± 0	0	0	0
4	10 ± 2	69 ± 2	9 ± 2	33 ± 7	1.0 ± 0.4
12	0.4 ± 0.1	48 ± 4	17 ± 0	67 ± 5	4 ± 0
24	0	36 ± 4	16 ± 1	64 ± 1	4 ± 0

Table S9 | HER activity of 1:1 H_2O:MeCN photocatalytic transfer hydrogenation system with and without nitrobenzene. Reaction conditions are 4MBA (100 mM, 1 mL) as proton and electron donor with or without 20 mM nitrobenzene as substrate under AM 1.5G irradiation at room temperature and pressure for 24 hours. 20 mg of $^{Co}MoS_2$-CN_x was used for each of the reactions. H_2 was quantified using a gas chromatograph and 2% methane as internal standard. Reaction solutions contained 0.01 M H_2SO_4

**Time**	**H_2 (No nitrobenzene) / µmol**	**H_2 (nitrobenzene) / µmol**
0	0	0
4	1.9 ± 0.4	0.03 ± 0.00
12	6.5 ± 0.1	0.04 ± 0.01
24	10.4 ± 0.3	1.4 ± 0.2

Table S10 | Photocatalytic transfer hydrogenation of nitrobenzene (20 mM) comparing H_2, Water and 4MBA as donors. Experiments were performed under simulated AM 1.5G irradiation at room temperature and pressure for 24 hours using 20 mg of $^{Co}MoS_2$-CN_x in 1:1 H_2O:MeCN under N_2. Products were determined using quantitative ^{1}H NMR with 0.75 wt% trimethylsilylpropionic acid (TSP) in D_2O as internal standard. Reaction solutions contained 0.01 M H_2SO_4.

**Time**	**0 hours**	**4 hours**		**24 hours**	
**H Donor**	**Nitrobenzene Conc. / mM**	**Nitrobenzene Conc. / mM**	**Aniline Conc. / mM**	**Nitrobenzene Conc. / mM**	**Aniline Conc. / mM**
4MBA	23	10 ± 2	9 ± 2	N.D.	16 ± 1
H_2	24	22 ± 1	N.D.	20 ± 0.0	N.D.
Water	22	24 ± 1	N.D.	24 ± 0.0	N.D.

Table S11 | Photocatalytic transfer hydrogenation of nitrobenzene (20 mM) under different reaction atmospheres. Experiments were performed under simulated AM 1.5G irradiation at room temperature and pressure for 24 hours using 20 mg of $^{Co}MoS_2$-CN_x in 1:1 H_2O:MeCN. Products were determined using quantitative ^1H NMR with 0.75 wt% trimethylsilylpropionic acid (TSP) in D_2O as internal standard. Reaction solutions contained 0.01 M H_2SO_4

**Time**	**4 hours**		**24 hours**	
**Reaction Atmosphere**	**4MBAd Conc. / mM**	**Aniline Conc. / mM**	**4MBAd Conc. / mM**	**Aniline Conc. / mM**
Under N_2	33 ± 7	9 ± 2	64 ± 1	16 ± 1
Under Air	59 ± 1	0.1 ± 0.07	82 ± 2	14 ± 2

Table S12 | Comparison of photocatalytic transfer hydrogenation of nitrobenzene (20 mM) activity using $^{Co}MoS_2$-CN_x and Pt-CN_x. Experiments were performed under simulated AM 1.5G irradiation at room temperature and pressure for 24 hours using 20 mg of catalyst in 1:1 H_2O:MeCN as solvent. Products were determined using quantitative ^1H NMR with 0.75 wt% trimethylsilylpropionic acid (TSP) in D_2O as internal standard. Reaction solutions contained 0.01 M H_2SO_4

**Time**	**4 hours**			**24 hours**		
**Photocatalyst**	**H_2 / µmol**	**4MBAd Conc. / mM**	**Aniline Conc. / mM**	**H_2 / µmol**	**4MBAd Conc. / mM**	**Aniline Conc. / mM**
$^{Co}MoS_2$-CN_x	~ 0	33 ± 7	9 ± 2	1.4 ± 0.2	64 ± 1	16 ± 1
Pt-CN_x	17 ± 5	48 ± 11	11 ± 2	57 ± 2	86 ± 2	10 ± 1

Table S13 | Photocatalytic transfer hydrogenation of nitrobenzene (20 mM) comparing waste substrates as donors. Experiments were performed under simulated AM 1.5G irradiation at room temperature and pressure for 24 hours using 20 mg of $^{Co}MoS_2$-CN_x in 1:1 H_2O:MeCN under N_2. Products were determined using quantitative ^1H NMR with 0.75 wt% trimethylsilylpropionic acid (TSP) in D_2O as internal standard. Reaction solutions contained 0.01 M H_2SO_4

Waste Donor	Aniline Conc. / mM
Glucose	14 ± 2
EG	7 ± 1
Glycerol	8.4 ± 0.1
TEOA	20.1 ± 0.1

Table S14 | Comparison of photocatalytic transfer hydrogenation under different light sources sing PET hydrolysate (8× diluted) containing EG (~ 173 mM) as donor. Experiments were performed without external heating at room temperature and pressure for 24 hours using 20 mg of $^{Co}MoS_2$-CN_x in 1:1 aqueous H_2O:MeCN under N_2. Products were determined using quantitative ^1H NMR with 0.75 wt% trimethylsilylpropionic acid (TSP) in D_2O as internal standard.

Time	Aniline yield / mM	H_2 / μmol	CO/ μmol
AM 1.5G 1 Sun	9 ± 2	0.08 ± 0.01	1.0 ± 0.1
AM 1.5G 3 Suns	15 ± 2	0.2 ± 0.2	1 ± 1
AM 1.5G 5 Suns	17 ± 1	0.3 ± 0.1	1.5 ± 0.3
LEDs (405 nm)	20 ± 1	11 ± 1	1.5 ± 0.2

Table S15 | Photocatalytic transfer hydrogenation of nitrobenzene to aniline using acid hydrolysates of condensation polymers as hydrogen and electron donors. Experiments were performed under 405 nm LED irradiation at room temperature and pressure for 24 hours using 20 mg of catalyst in 1:1 H_2O:MeCN as solvent under N_2. Products were determined using quantitative ^{1}H NMR with 0.75 wt% trimethylsilylpropionic acid (TSP) in D_2O as internal standard.

Plastic	Aniline Conc. / mM	Aniline yield / %
PET	19 ± 2	> 99
Nylon 66	17 ± 1	76.0 ± 0.3
Polyurethane	21.0 ± 0.3	> 99

Table S16 | Hydrogen (H_2) and carbon monoxide (CO) evolution experiments in 4 hours from photoreforming of glycolaldehyde at different pH. 100 mM of glycolaldehyde was added to a solution of 1:1 H_2O:MeCN. Reaction was conducted at room temperature and pressure under 405 nm LED irradiation using $^{Co}MoS_2$-CN_x. Gaseous products were quantified using a gas chromatograph with 2% methane as internal standard.

Substrate	H_2 Conc. / μmol	CO Conc. / μmol
Glycolaldehyde pH 0	30	19
Glycolaldehyde pH 2	21	7

Table S17 | Transfer hydrogenation of nitrobenzene to aniline using hydrolysates mimics as hydrogen and electron donors. Experiments were performed under 405 nm LED irradiation at room temperature and pressure for 24 hours using 20 mg of using $^{Co}MoS_2$-CN_x. in 1:1 hydrolysate: acetonitrile as solvent under N_2. Products were determined using quantitative ^{1}H NMR with 0.75 wt% trimethylsilylpropionic acid (TSP) in D_2O as internal standard.

Hydrolysate / Mimic	Aniline yield / mM
1 M KOH	~0
H_2O	3 ± 1
PET Hydrolysate	19 ± 2

Table S18 | Comparison of PTH activity of $^{Co}MoS_2$-CN_x under monochromatic 405 nm irradiation for various exclusion controls scenarios. PTH does not occur in the absence of light, photocatalyst, nitro substrate or plastic hydrolysate. Products were determined using quantitative ^{1}H NMR with 0.75 wt% trimethylsilylpropionic acid (TSP) in D_2O as internal standard.

Time	Aniline yield / mM	H_2 / µmol
No Light	N.D.	N.D.
No Photocatalyst	N.D.	N.D.
No $^{Co}MoS_2$	N.D.	N.D.
Only $^{Co}MoS_2$	N.D.	N.D.
$^{Co}Mo(O)_y$-gCN_x	N.D.	N.D.
No Nitrobenzene	N.D.	N.D.
No hydrolysate	N.D.	N.D.
Mechanically mixed $^{Co}MoS_2$ + CN_x	12 ± 3	N.D.
$^{Co}MoS_2$-CN_x	19 ± 2	11 ± 1

Table S19 | Cost price of hydrolysis, photoreforming and transfer hydrogenation products

Product	Price / £ kg^{-1}	Ref
Ethylene Glycol	0.8	[14]
Terephthalic Acid	0.9	[4 15]
Acetic Acid	1 – 1.3	[415]
Formic Acid	0.4 – 0.5	[415]
Hydrogen (SMR)	0.8	[16]

Table S20 | Consumables cost for acid photoreforming and photocatalytic transfer hydrogenation of 9 tons PET per day

**Consumable**	**Price / £ kg^{-1}**	**Amount needed / kg**	**Total cost / £**	**Ref**
**Photocatalyst (900 kg of $^{Co}MoS_2$-CN_x, assumed biweekly usage)**				
Melamine	1	64	77	[17]
Molybdenum(VI) Oxide (MoO_3)	12	2	25	[4]
Cobalt(II,III) Oxide (Co_3O_4)	20	1	22	[18]
Potassium thiocyanate (KSCN)	1	77	62	[19]
Consumable	Price / £ L^{-1}	Amount needed / L	Total cost / £	Ref
**7.5 M H_2SO_4 (9000 L)**				
Water for dilution	See table S27	33750		
Sulfuric acid, 98%[a]	0.2	1172	188[a]	[20]
Acetonitrile + 10% makeup	13	136[b]	1800	[21]

[a] H_2SO_4 is changed biweekly
[b] Acetonitrile recycled throughout the year with 10% annual makeup

Table S21 | Consumables cost for Industrial Hydrogenation

**Consumable**	**Price / £ L^{-1}**	**Amount needed / L**	**Total cost / £**	**Ref**
Water for dilution	See table S27			
Methanol + 50% makeup[a]	0.5	54	27	[22]
Formaldehyde	0.8	0.8	0.6	[23]

[a] Methanol recycled throughout the year with 50% annual makeup

Table S22 | Catalyst cost for Industrial Hydrogenation (Yearly)

**Consumable**	**Price / £ kg^{-1}**	**Amount needed / kg**	**Total cost / £**	**Ref**
Activated Carbon	3	6	17	[24]
Palladium Chloride (60% Pd)[a]	15000	0.3	4200	[25]
Sodium Carbonate	1	0.2	0.2	[26]

Sodium Chloride	0.2	0.2	0.1

[a] The lower bound for $PdCl_2$ is the value of Pd metal it contains.

Table S23 | Energy cost for Acid PR of 9 tons PET per day

Consumable	Estimated Consumption / kWh	Unit Cost / £ kWh^{-1}	Total cost / £ day^{-1}
Acid Hydrolysis[a]	2056[b]	0.2	329
Light irradiation	Solar	-	-

[a] Electricity cost was taken to be £0.16/kWh[15]

[b] Calculated using $Q_{tot} = Q_{heat} + Q_{conv}$ where $Q_{heat} = c_p m\Delta T$ (C_p = 3000 J kg^{-1} C^{-1}, ΔT = 140 °C – 25 °C) and $Q_{conv} = \frac{kA\Delta Tt}{d}$ ($k = 0.5$ W m^{-1} K^{-1} for glass lining, A = cross-sectional area of steel tank = 16.6 m^2, ΔT = 140 °C – 25 °C, d = material thickness = 10 mm, t = 86,400s)

Table S24 | Energy cost for industrial hydrogenation

Consumable	Estimated Consumption / kWh	Unit Cost / £ kWh^{-1}	Total cost / £ day^{-1}
Hydrogen compression (60 bar)[a]	128	0.2	21
Temperature control[b]	188	0.2	30

[a] Calculated using estimated consumption = m_{H2} [kg/day] × W_{comp} [kWh/kg], W_{comp} [kWh/kg] = $\frac{\left(\frac{1000}{2.016}\right)RTln(\frac{P_2}{P_1})}{\eta \times 3.6 \times 10^6}$, where R = 8.314 J $mol^{-1}K^{-1}$, η = 0.70

[b] Calculated using assumptions heat from 25 °C → 50 °C (ΔT = 25 K), Methanol ρ = 0.792 kg L^{-1}, c_p= 2.5 kJ kg^{-1} K^{-1} (25–50 °C), Hydrogen c_p= 14.3 kJ kg^{-1} K^{-1} (near ambient). Therefore $Q_{Methanol}$ = 10479 × 2.5 × 25 = 654885 kJ (654.9 MJ), Q_{H2} = 64 × 14.3 × 25 = 22,880 kJ (22.9 MJ), and Q_{total} = 654.9 + 22.9 = 677.8 MJ day^{-1} = 188.3 KWh day^{-1}

Table S25 | Estimation of hydrogenation equipment costs based on approximations from Towler & Sinott

**Equipment**	**Sizing basis[27-29]**	**Base (2010, CS)**	**2019 (CS)**	**FBM[a]**	**Installed (USD)**	**Installed (GBP)**
Trickle-bed reactor (vertical, packed)	Vessel 0.8 m^3 (LHSV ~1 h^{-1} on 0.55 m^3 bed)[b]	\$50k × $(0.8)^{0.6}$ = \$43.8k	\$50k	6	\$275k	£220k
Condenser (S&T)	A = 15 m^2 (Q ~100 kW; U 500; ΔT_lm 15 K)	\$20k × $(15/10)^{0.6}$ = \$26k	\$29k	Included in plant installation costs		
KO drum (vertical)	Vessel 1.0 m^3	\$30k	\$34k			

[a] Refers to bare-module factors that accounts for pressure rating corrections, foundations and assembly[27-29]

[b] Liquid hourly space velocity (LHSV) is the inverse of reactor space time

Table S26 | Values used for assessing photoreforming and photocatalytic transfer hydrogenation of nitrobenzene

Component	Cost / £	Per unit	Quantity	Total Cost / £	Ref
	**Capital**				
PVC cell chamber + assembly labor	53	m²	9000	474300	
Plexiglass cover	30	m²	9000	270000	
Support frame	23	m²	9000	202500	
Pump	3000	unit	30	90000	
PTFE-lined tubing (acid service)	7	m	9000	62100	[41530]
Glass-lined carbon steel container (pre-treatment, 7.5 M H_2SO_4)	1798	m^3	15	26970	
HDPE tank (dilute hydrolysate storage)	205	m³	38	7790	
PTFE filter (acid compatible, 5 µm)	30	m²	15	456.8	
Polypropylene container for Nitrobenzene storage	1700	m^3	15	25500	[15]
Polypropylene container for Aniline storage	1700	m^3	15	25500	[15]
405 nm “purple” LED arrays	2	m²	9000	19800	[431]
Control systems	4	m²	9000	36900	
Installation labor	23	m²	9000	202500	[32]
Other system costs	43	m²	9000	387000	
Planning & consulting	add 10% of capital cost	—	+ 10%	183132	
Administration & insurance	add 2% of capital cost	—	+ 2%	36626	[33]
Contingency planning	add 15% of capital cost	—	+ 15%	274698	
Interest on investment			+ 10% p.a.	183132	[34]
**Total**				**2508904**	
	**Daily Operation**				
Operating labour	15	h	4 h day^{-1}	60	[15]
Energy for acid hydrolysis (heating)[a]	329	day		329	[4]
Energy for pumps (0.23 kW/h)	0.04	h	24 h	1	[15]
Water treatment	0.002	L	9000 L	14	[34]
Waste disposal (solid plastic residue)	0.02	kg	900 kg	20	[15]
Maintenance & repair	add 5% of fixed capital costs	—		17	[1532]
Miscellaneous expenses	+ 5 %	—		22	[1532]
**Total**				**463**	
**Total (operation over 20-year span)**				**3382367**	
	**Daily Consumables**				
$^{Co}MoS_2$–CN_x photocatalyst[b]	0.2	kg day^{-1}	900	180	
99% H_2SO_4[b]	0.2	L day^{-1}	1172	188	
H_2O[b]	0.002	L	33750	51	
Acetonitrile[b]				1800	
Nitrogen (sparge)	0.004	L	30000	120	
Nitrobenzene	1.2	kg	1323	1588	
Plastic waste feed (gate fee)	-0.05[c]	kg	9000	-450	
**Total**				**3476**	
**Total (Consumables over 20-year span)**				**25376260**	
**Total Cost**				**31267531**	
	**Daily Output**				
Aniline		kg	1000		
Acetic Acid	1.3	kg	1742	2265	
Formic Acid	0.5	kg	148	74	
TPA	0.9	kg	6100	5490	
Total Aniline (Output over 20-year span)			7300000		
Total Acetic Acid (Output over 20-year span)				16531580	
Total Formic Acid (Output over 20-year span)				540200	
Total TPA (Output over 20-year span)				40077000	
**Total (Output over 20-year span)**				**57148780**	
**Production cost (LCOA)**				**4.3**	
**Production cost (with sale of TPA)**				**-1.2**	
**Production cost (with sale of TPA, Acetic Acid and Formic Acid)**				**-3.5**	

[a] Calculated in Table S23

[b] Calculated in Table S20

[c] The plant would be paid a gate fee for taking plastic waste, hence the negative cost.

Table S27 | Values used for assessing industrial hydrogenation of nitrobenzene

Component	Cost / £	Per unit	Quantity	Total Cost / £	Ref
**Capital**					
Hydrogenation reactor (trickle-bed)[a]	Estimated using the log-linear equipment sizing correlations of Towler & Sinott (Table S7)[27]			220000	[27-29]
Hydrogen recycle compressor	40000	unit	2	80000	[41534]
Hydrogen storage	2000	kg H_2	45	90000	
Condensers[a]	29100	15 m^2	15	29100	[27-29]
Pump	3000	Unit	30	90000	
Polypropylene container for Nitrobenzene storage	1700	m^3	15	25500	[41530]
Polypropylene container for Aniline storage	1700	m^3	15	25500	
Gas-liquid separator/knockout drum[a]	34200	m^3	1	34200	[27-29]
Instrumentation and Control	add 40% of capital cost		+ 40%	237720	
Installation labor	add 40% of capital cost		+ 40%	237720	
Piping and Electrical systems	add 40% of capital cost		+ 40%	237720	[13]
Planning & consulting	add 10% of capital cost		+ 10%	59430	
Other system costs	add 25% of capital cost		+ 25%	148575	
Service facilities	add 70% of capital cost		+ 70%	416010	
Administration & insurance	add 2% of capital cost		+ 2%	59430	[33]
Contingency planning	add 15% of capital cost		+ 15%	89145	
Interest on investment			+ 10% p.a.	59430	[34]
**Total**				**2139480**	
**Daily Operation**					
Operating labour	15	h	4 h day-1	60	[15]
Energy for temperature and systems control	30	day		30	
Energy for pumps & compressors (0.23 kW/h)	21	day		21	[15]
Steam for reboilers[b]	0.03	day		0.03	
Cooling water[b]	0.002	day		0.002	
Water treatment	0.002	L	3000 L	5	
Waste disposal (tar)	0.02	kg	1000 kg	22	[15]
Maintenance & repair	add 5% of fixed capital costs			15	[1532]
Miscellaneous expenses	+ 5 %			8	[1532]
**Total**				**160**	
**Total (operation over 20-year span)**				**1165731**	
**Daily Consumables**					
Activated carbon[c]	2.6	kg	0.02	0.04	
Palladium chloride (60% Pd)[c]	15000	kg	0.0008	12	
Sodium Carbonate[c]	1	kg	0.0004	0.0004	
Sodium Chloride[c]	0.2	kg	0.0005	0.0001	
Formaldehyde[c]	0.8	L	0.8	0.002	
Methanol[c]	0.5	L	0.2	0.1	
Nitrobenzene	1.2	kg	1323	1588	
Hydrogen (SMR)	0.84	kg	65	55	
**Total**				**1654**	
**Total (Consumables over 20-year span)**				**12073305**	
**Total Cost**				**15378515**	
**Daily Output**					
**Aniline**		kg	1000		
**Total Aniline (Output over 20-year span)**			7300000		
**Production cost (LCOA)**				**2.1**	

[a] Purchased costs were estimated using the log-linear equipment sizing correlations of Towler & Sinott (2019) (CS base), time-indexed using CEPCI (2010-2019) and corrected for SS-304 and 60 bar via material and pressure factors.[27]

[b] Calculated in Table S24

[c] Calculated in Table S25

Table S28 | Values used for assessing carbon emissions and footprint from photoreforming and photocatalytic transfer hydrogenation of nitrobenzene

Component	Carbon Emissions (kg CO_{2-eq})	Per unit	Ref	Quantity	Carbon Footprint (kg CO_2)
PVC cell chamber + assembly labor	17.6	m^2	[34-35]	9000 m^2	158400
Plexiglass cover	3.2	m^2	[34-35]	9000 m^2	28800
Support frame	19	m^2	[34-35]	9000 m^2	171000
PTFE-lined tubing (acid service)	9.6	kg	[36]	9000 kg	86400
Glass-lined carbon steel container (pre-treatment, 7.5 M H_2SO_4)	1.8	kg	[35]	52500 kg	94500
HDPE tank (dilute hydrolysate storage)	4.9	kg	[37]	45600 kg	223440
PTFE filter (acid compatible, 5 μm)	9.6	kg	[36]	4 kg	38
Polypropylene container for Nitrobenzene storage	1.6	kg	[38]	1240 kg	1984
Polypropylene container for Aniline storage	1.6	kg	[38]	1240 kg	1984
405 nm “purple” LED arrays	12.33[a]	kg	[3539]	19170 kg[a]	236366
Installation labor	+30%		[34]		300874
**Total**					**1303786**
**Daily Operation**					
Energy for acid hydrolysis (heating)[b]	0.12	kWh	[40]	2056 kWh	255
Energy for pumps (0.23 kW/h)	0.04	h	[34]	24 h	1.1
Water treatment	0.0003	L	[34]	9000 L	2.7
Waste disposal (solid plastic residue)	0.015	kg	[34]	900 kg	13.5
**Total**					273
**Total (20 years)**					**1992900**
**Daily Consumables**					
$^{Co}MoS_2$–CN_x photocatalyst[c]	18.8	kg	[41-43]	64.3 kg	1208.5
99% H_2SO_4	0.14	kg	[44]	1172 L	164.1
H_2O[b]	0.0003	L	[34]	33750 L	10.8
Acetonitrile[d]	1.4	kg	[45]	107 kg	149.8
Nitrogen (sparge)	0.0005	L	[34]	30000 L	16.2
Nitrobenzene[e]	2	kg	[46]	1323 kg	2646
Plastic waste feed (avoided incineration)[f]	-0.4	kg	[47]	9000 kg	-3879
**Total**					316.3
**Total (20 years)**					**2309282**
**Daily Output**					
Aniline				1000	
**Total Aniline (Output over 20-year span)**				**7300000**	
**Carbon Footprint**					**0.8**

[a] One LED array weighs 2.13 kg and is composed of aluminum. Carbon footprint was calculated based on carbon footprint of aluminum and LEDs. Aluminum carbon footprint was 12 kg CO_{2-eq}/kg with embodied energy of 200 MJ/kg whereas LEDs had a carbon footprint of 0.33 kg CO_{2-eq}/kg[3539]

[b] UK electricity carbon footprint estimated to be 0.124 kg CO_{2-eq}/kWh[40]

[c] Photocatalyst was calculated using a combination of melamine (5.42 kg CO_{2-eq}/kg)[41], MoO_3 (3.79 kg CO_{2-eq}/kg)[42], and Co_3O_4 (24 kg CO_{2-eq}/kg)[43]. No carbon footprint data was obtained for KSCN therefore the carbon footprint was estimated using NH_4SCN + KOH and was 5 kg CO_{2-eq}/kg.

[d] Acetonitrile is assumed to be bio-based with a carbon footprint of 1.4 kg CO_{2-eq}/kg[45]

[e] Estimated from ecoinvent 36 database reports[46]

[f] Carbon footprint due to CO_2 emissions avoided from upcycling rather than incineration and landfills

Table S29 | Values used for assessing carbon emissions and footprint from industrial hydrogenation of nitrobenzene

Component	Carbon Emissions (kg $CO_{2\text{-eq}}$)	Per unit	Ref	Quantity	Carbon Footprint (kg CO_2)
Hydrogenation reactor (trickle-bed)	7.1[a]	kg	[48]	1800 kg	12852
Hydrogen recycle compressor	1.8[b]	kg	[35]	2600 kg	4680
Hydrogen storage[c]	20	kg	[34]	1400 kg	28000
Condenser[d]	2.8	kg	[36]	700 kg	1960
Polypropylene container for Nitrobenzene storage	1.6	kg	[38]	1240 kg	1984
Polypropylene container for Aniline storage	1.6	kg	[38]	1240 kg	1984
Gas-liquid separator/knockout drum[e]	6.8	kg	[48]	1100 kg	7480
Installation labor	+50%		[34]		29470
**Total**				88410	
**Daily Operation**					
Energy for temperature and systems control	0.12	kWh	[40]	316.3 kWh	39.2
Energy for pumps & compressors (0.23 kW/h)	0.04	h	[34]	24 h	1.1
Water treatment	0.0003	L	[34]	3000 L	0.9
Waste disposal (tar)	0.02	kg	[34]	1000 kg	15
**Total**					56.2
**Total (20 years)**					410114
**Daily Consumables**					
Activated carbon	1.3[f]	kg	[24]	0.02 kg	0.02
Palladium chloride (60% Pd)	6800[g]	kg	[49-50]	0.0008 kg	5.2
Sodium Carbonate	0.5	kg	[51]	0.0004 kg	0.00018
Sodium Chloride	0.1	kg	[52]	0.0005 kg	0.00003
Formaldehyde	1.0	kg	[53]	0.8 kg	0.8
Methanol	2.8	kg	[54]	0.2 kg	2.9
Nitrobenzene	2	kg	[46]	1323 kg	2646
Hydrogen (SMR)[h]	9.4	kg	[55]	65 kg	608
**Total**					**3263**
**Total (20 years)**					**23817646**
**Daily Output**					
Aniline				1000	
**Total Aniline (Output over 20-year span)**				**7300000**	
**Carbon Footprint**					**3.3**

[a] Estimated based on stainless steel containing 30% scrap (6.8 kg $CO_{2\text{-eq}}$/kg) + assumed 5% for fabrication add-ons[48]

[b] Estimated using carbon steel[35]

[c] H_2 assumed to be compressed at 700 bar equivalent to approx. 1.95 kg H_2 per cylinder (45 units proposed)[56]

[d] Estimated to contain 80% Carbon steel and 20% stainless steel, carbon footprint = 0.8*1.8 + 0.2*6.8 = 2.8 kg $CO_{2\text{-eq}}$/kg

[e] Stainless steel (6.8 kg $CO_{2\text{-eq}}$/kg)[48]

[f] Activated carbon utilized is based on coconut shell route and given as 1.26 kg $CO_{2\text{-eq}}$/kg[24]

[g] Derived from BAFA/ecoinvent Pd metal = 11 289 kg CO_2e kg^{-1} [49] × 0.6 + Cl_2 ≈ 1 kg CO_2e kg^{-1} [50]

[h] H_2 produced from steam methane reforming

Table S30 | Summary of Technoeconomic and Environmental Analysis

**Parameter**	**Photocatalytic Transfer Hydrogenation**	**Hydrogenation**
Hydrogen Source	Waste plastics (EG)	Methane (SMR)
Electrons Source	Waste plastics (EG)	Molecular Hydrogen
Energy Source for hydrogenation	Solar	Electric
Feed / ton year^{-1}	3285 tons of PET Waste	24 tons of H_2
Capital Costs / £ year^{-1}	125,445	106,974
Operational Costs / £ year^{-1}	169,118	58,287
Consumable Costs/ £ year^{-1}	1,268,813	603,665
Levelized cost of Aniline (LCOA) / £ kg AN^{-1}	**4.3**	**2.1**
Carbon footprint / kg CO_2	**0.8**	**3.3**
Co-products	TPA, Acetic Acid, Formic Acid	Tar
Revenue from co-products / £ year^{-1}	1,294,062	—

Table S31 | Summary of Levelized Cost of Aniline and Carbon Footprint

**Scenario**	**LCOA, £ kg^{-1} AN**	**Carbon Footprint, kg $CO_{2\text{-eq}}$ kg^{-1} AN**
Conventional Pd/C hydrogenation	2.1	3.3
PTH using plastics	4.3	0.8
PTH with 50% less LEDs and photocatalyst cost	4.0	0.1
PTH with 80% less acetonitrile	2.9	0.7

Supplementary Figures

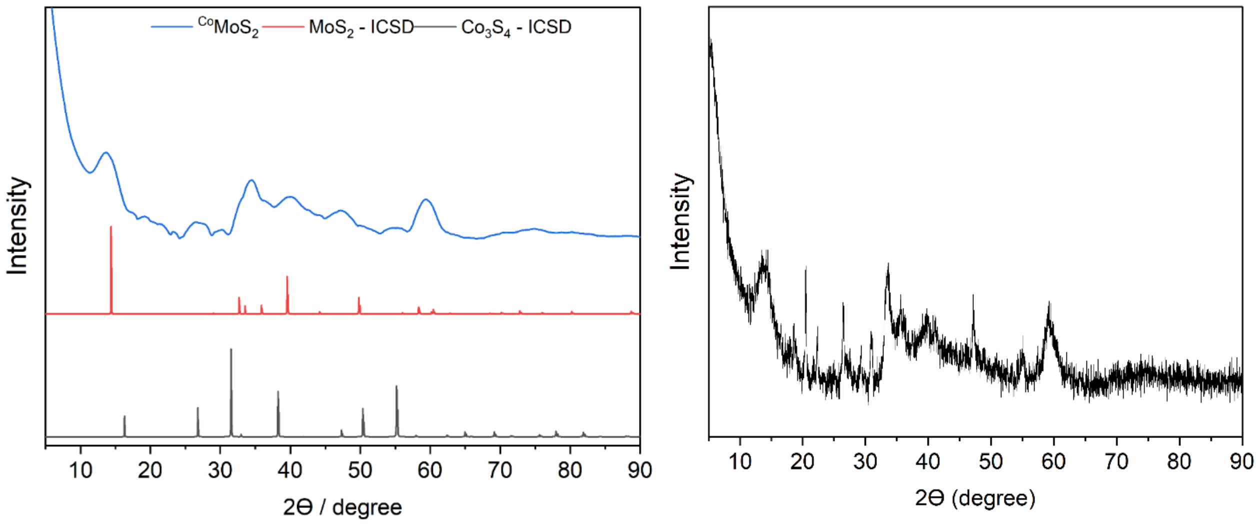

Fig. S1 | Powder XRD pattern of cobalt promoted molybdenum sulfide ($^{Co}MoS_2$) compared with reference MoS_2 and Co_3S_4 ICSD patterns. On the right shows raw unsmoothed pattern of $^{Co}MoS_2$. Broad features aligned with MoS_2 and Co_3S_4 reflections indicate the likely presence of these phases in a poorly crystalline, highly disordered form. These broad features also indicate the possibility of MoS_x and Co_xS_y phases within the structure.

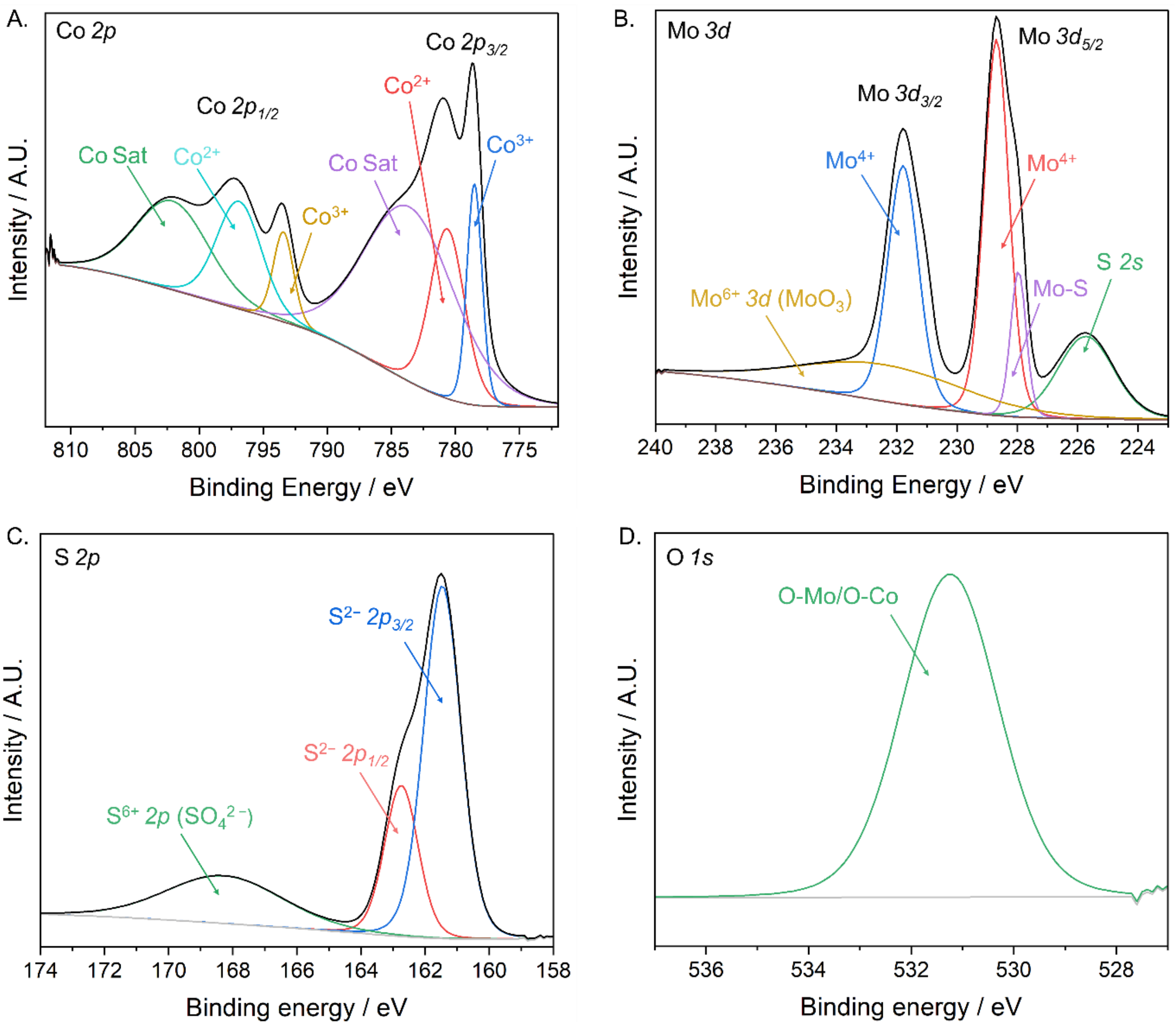

Fig. S2 | XPS spectra of $^{Co}MoS_2$ showing (a) Co 2p, (b) Mo 3d, (c) S 2p, (d) O 1s regions. The Co 2p spectrum revealed mixed Co^{2+}/Co^{3+} oxidation states and associated satellite peaks (~778, ~780 eV), confirming the presence of mixed cobalt sulfides. The Mo 3d spectrum showed reduction of Mo^{6+} ($3d_{3/2}$ at 232 eV) to Mo^{4+} ($3d_{5/2}$ at ~228 eV), consistent with MoS_2 formation.[6] This was supported by the S 2p region, which displayed a $2p_{3/2}$ peak at ~161 eV, characteristic of S^{2-} in metal sulfides.[6]

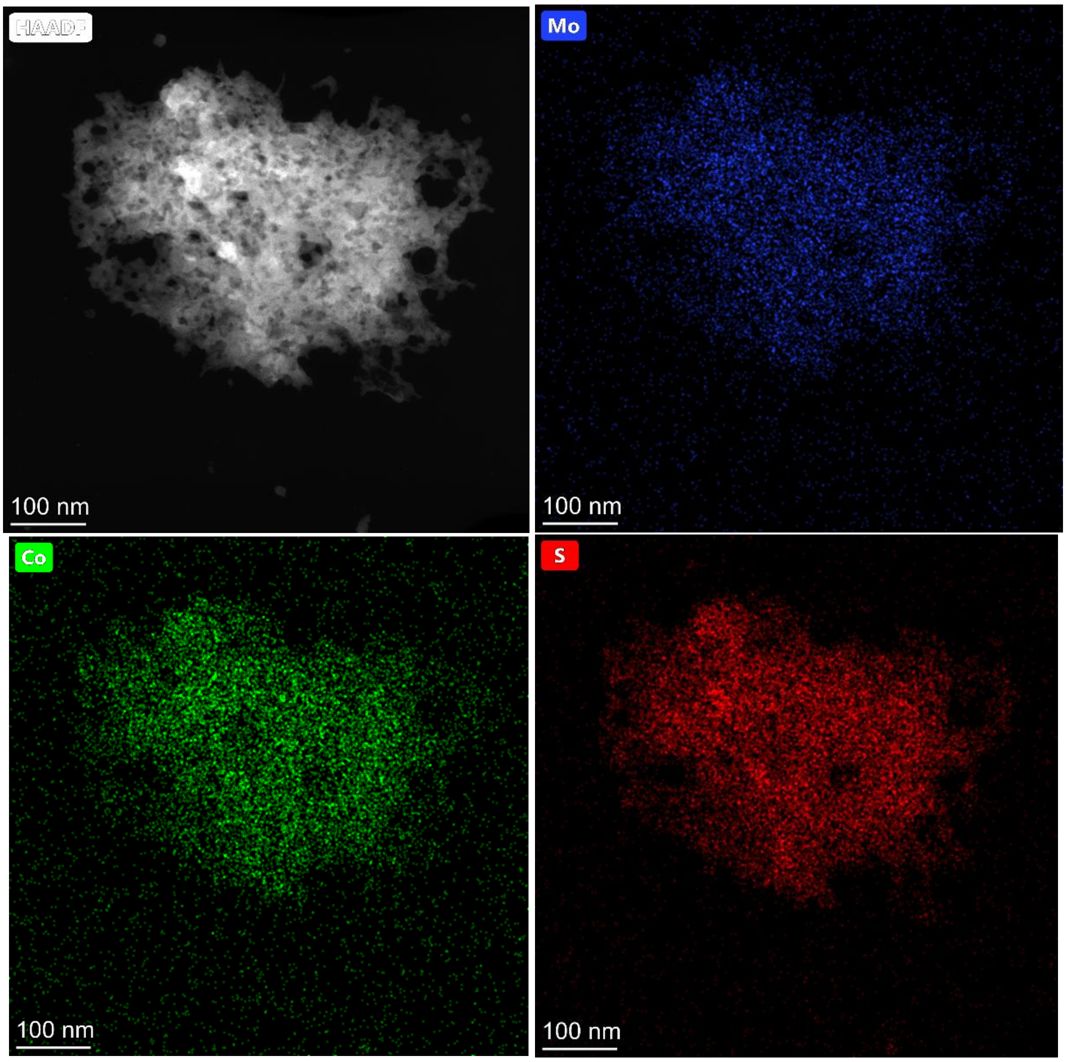

Fig. S3 | High-Angle Annular Dark-Field Scanning Transmission Electron Microscopy (HAADF-STEM) image with EDX maps of Mo, Co, and S for $^{Co}MoS_2$. All elements are homogeneously dispersed, indicating a well-mixed, compositionally uniform Co-Mo-S phase.

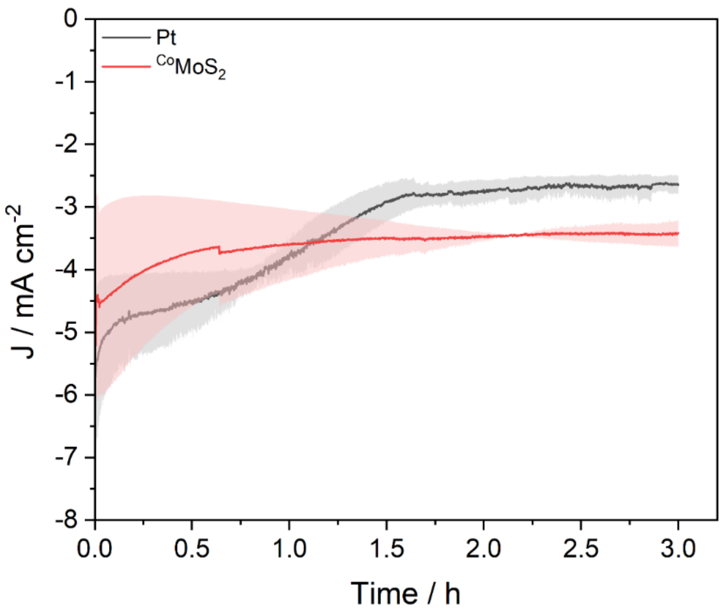

Fig. S4 | Chronoamperometric curves at -0.7 V vs RHE on NB reduction in a three-electrode cell (1:1 H_2O:MeCN; 0.1 M K_2SO_4; 0.01 M H_2SO_4). Working electrode was $^{Co}MoS_2$ ink dropcast on graphite foil (1 cm^2) with Pt mesh as counter. When using Pt mesh as working electrode, another Pt mesh was used as counter.

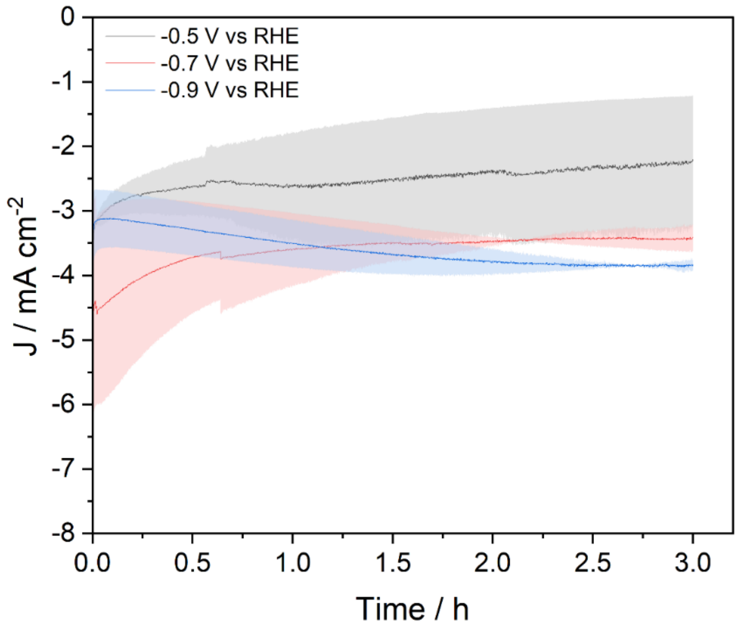

Fig. S5 | Chronoamperometric curves at various potentials vs RHE on NB reduction in a three-electrode cell (1:1 H_2O:MeCN; 0.1 M K_2SO_4; 0.01 M H_2SO_4) using $^{Co}MoS_2$ electrode. Working electrode was $^{Co}MoS_2$ ink dropcast on graphite foil (1 cm^2) with Pt mesh as counter.

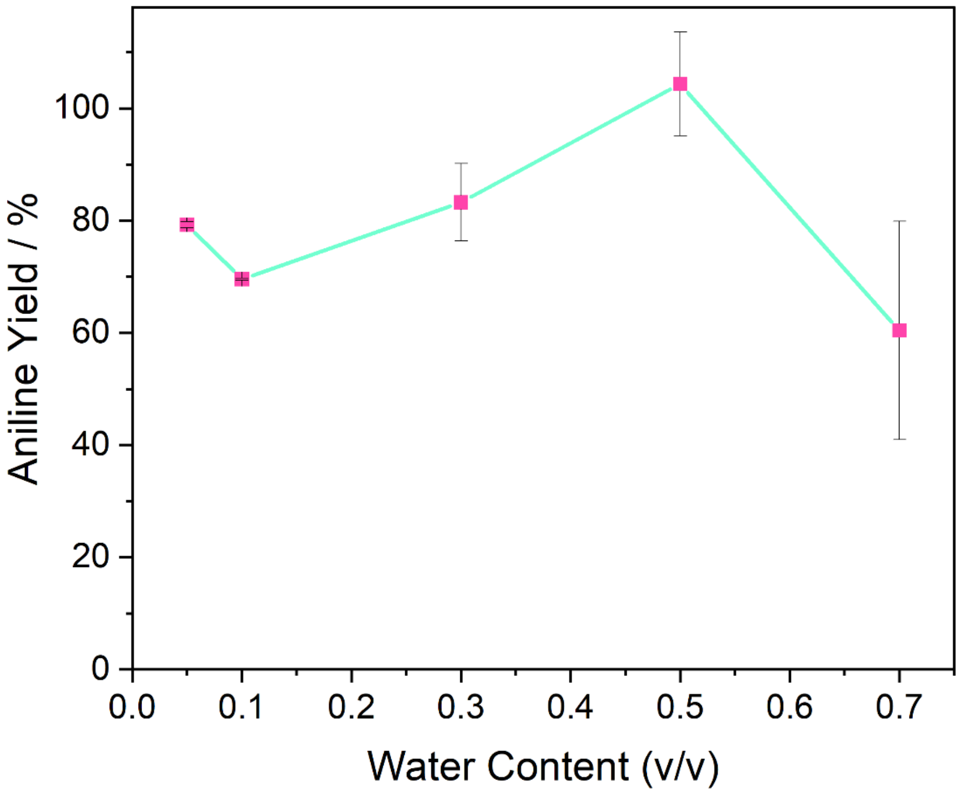

Fig. S6 | Solvent ratio (H_2O:MeCN) effect on photocatalytic activity and yield of aniline from nitrobenzene hydrogenation. 4MBA (100 mM, 1 mL) is used as proton and electron donor with 20 mM nitrobenzene as substrate under AM 1.5G irradiation at room temperature and pressure for 24 hours. 20 mg of catalyst was used for each of the reactions. Reaction solutions contained 0.01 M H_2SO_4.

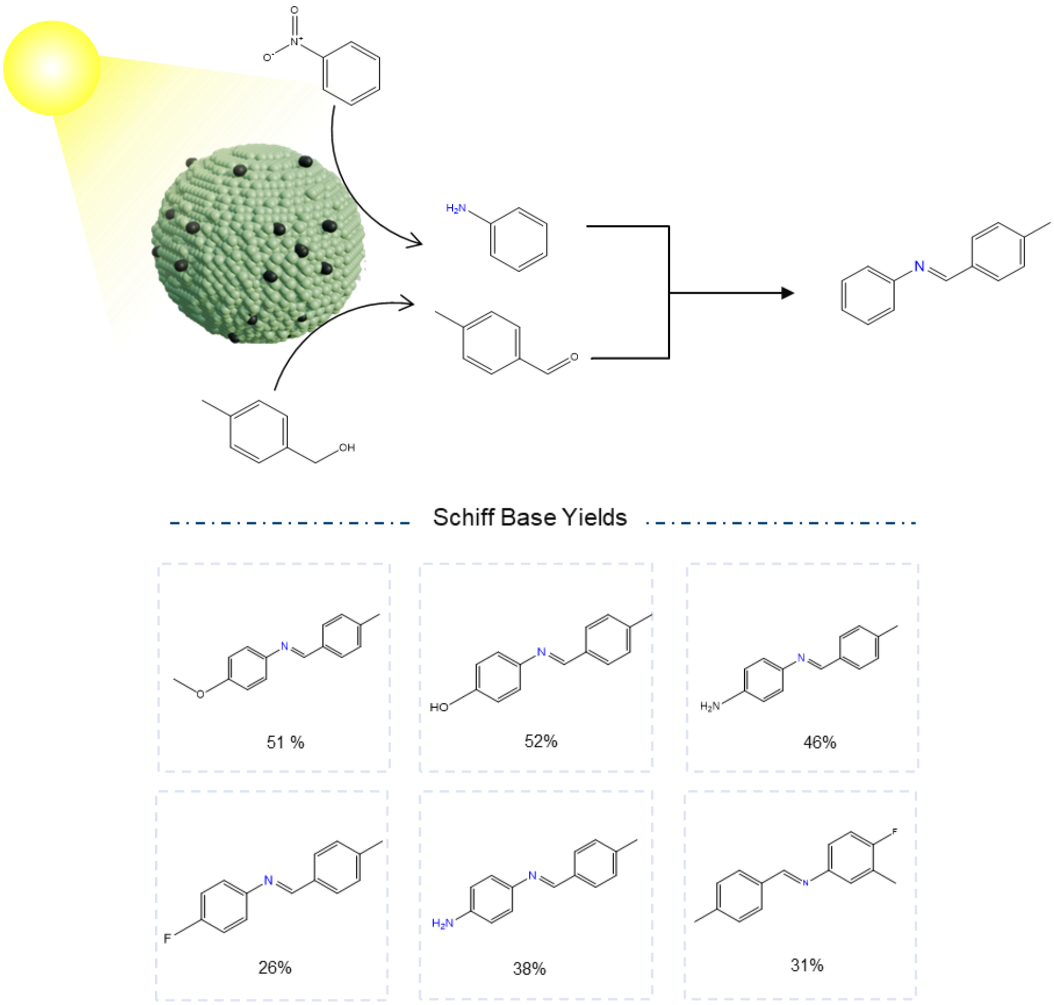

Scheme S1 | Formation of Schiff base from condensation of 4-methylbenzaldehyde, oxidation product of 4-methylbenzylalchol and aminoarenes obtained from transfer hydrogenation of nitroarenes

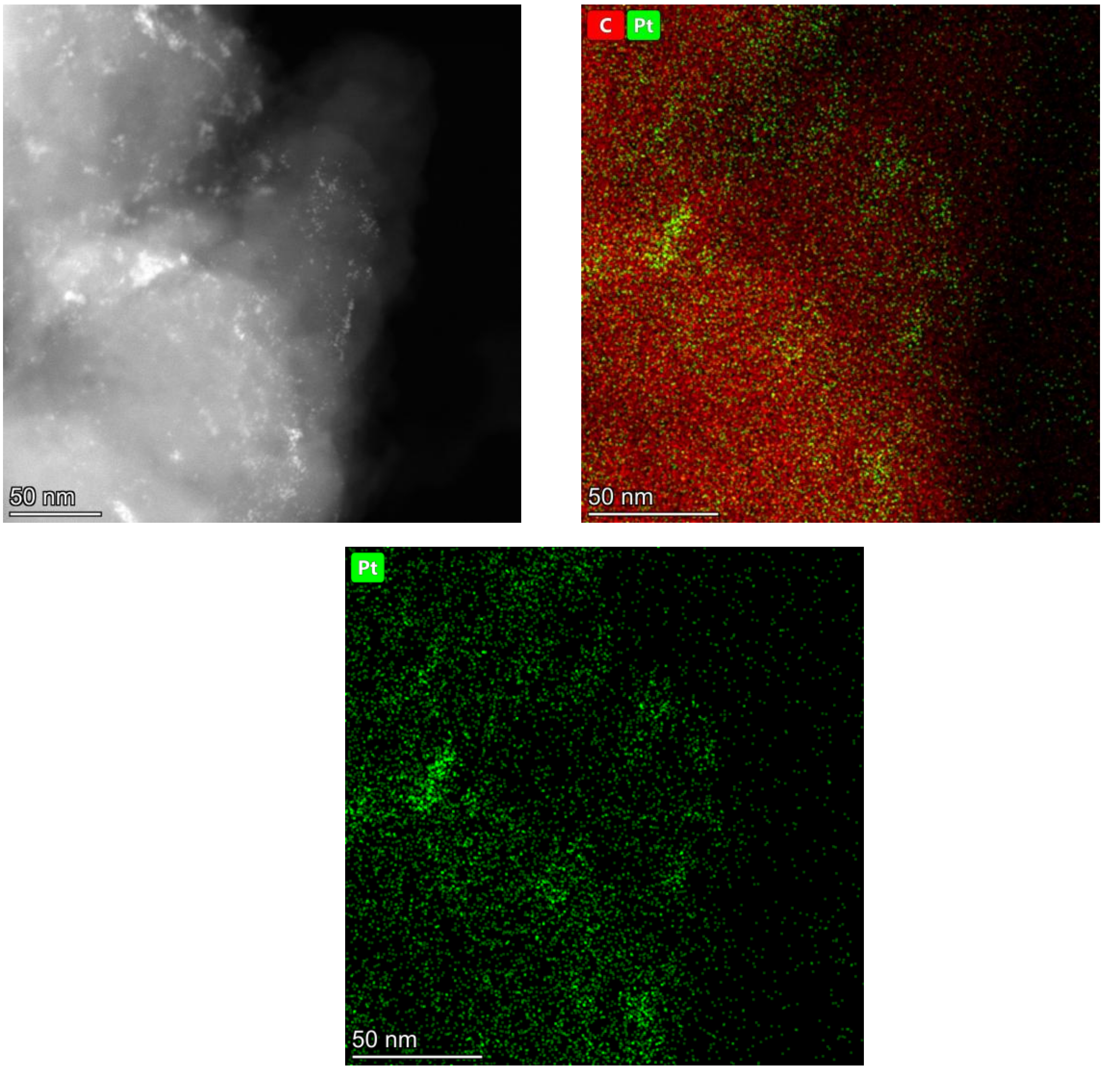

Fig. S7 | High-Angle Annular Dark-Field Scanning Transmission Electron Microscopy (HAADF-STEM) image with EDX maps of C and Pt for Pt-CN_x. Pt nanoparticles are homogeneously dispersed with CN_x.

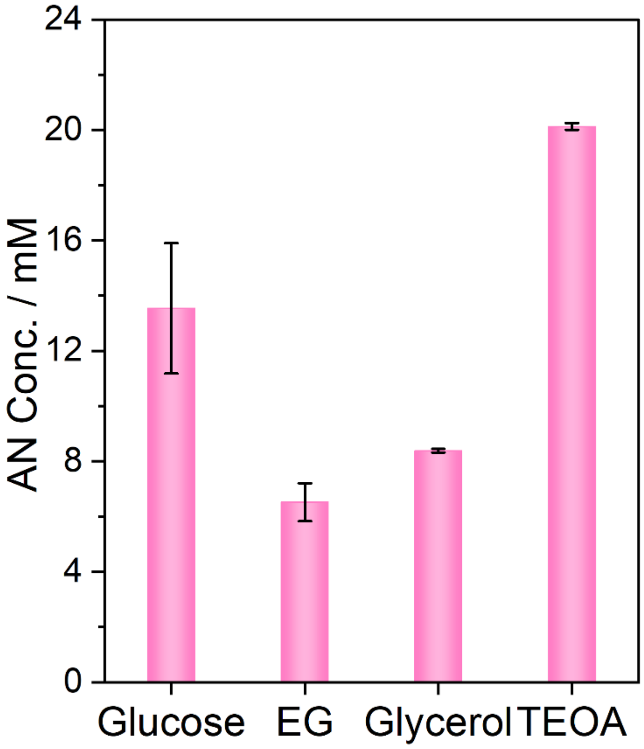

Fig. S8 | Photocatalytic transfer hydrogenation of nitrobenzene comparing model waste substrates as electron donors using AM 1.5G at 25 °C for 24 hours.

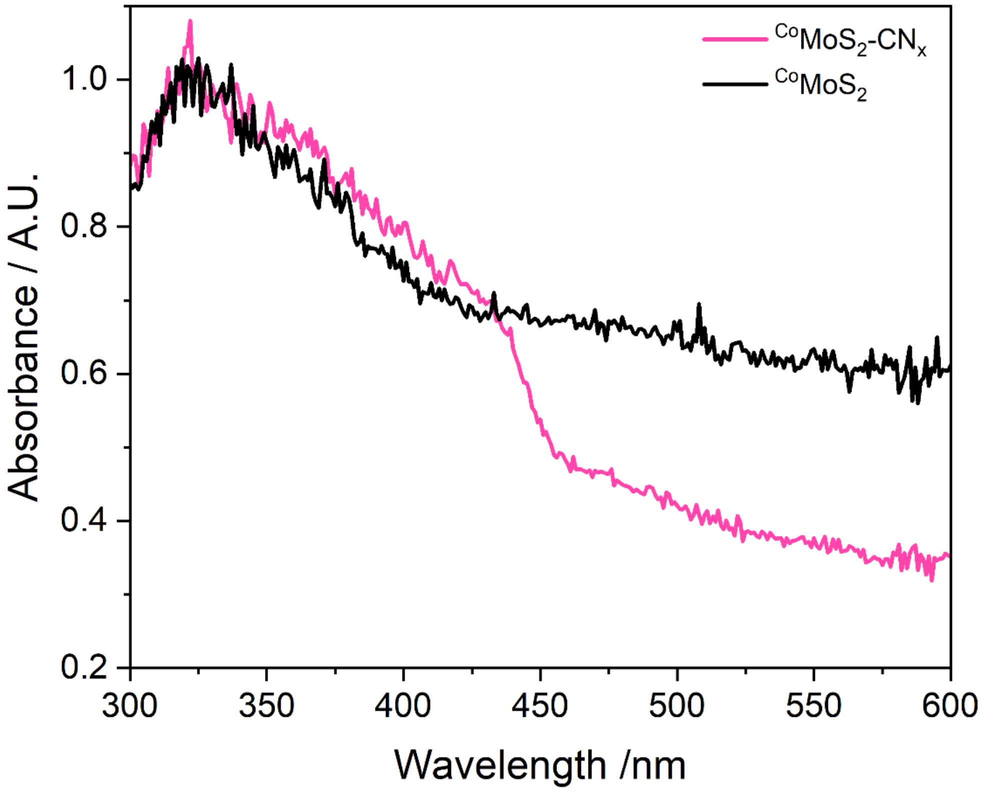

Fig. S9 | Diffuse-reflectance UV–Vis comparing $^{Co}MoS_2$ and $^{Co}MoS_2$-CN_x. $^{Co}MoS_2$-CN_x exhibits semiconductor behavior with maximum absorption between 300 and 450 nm

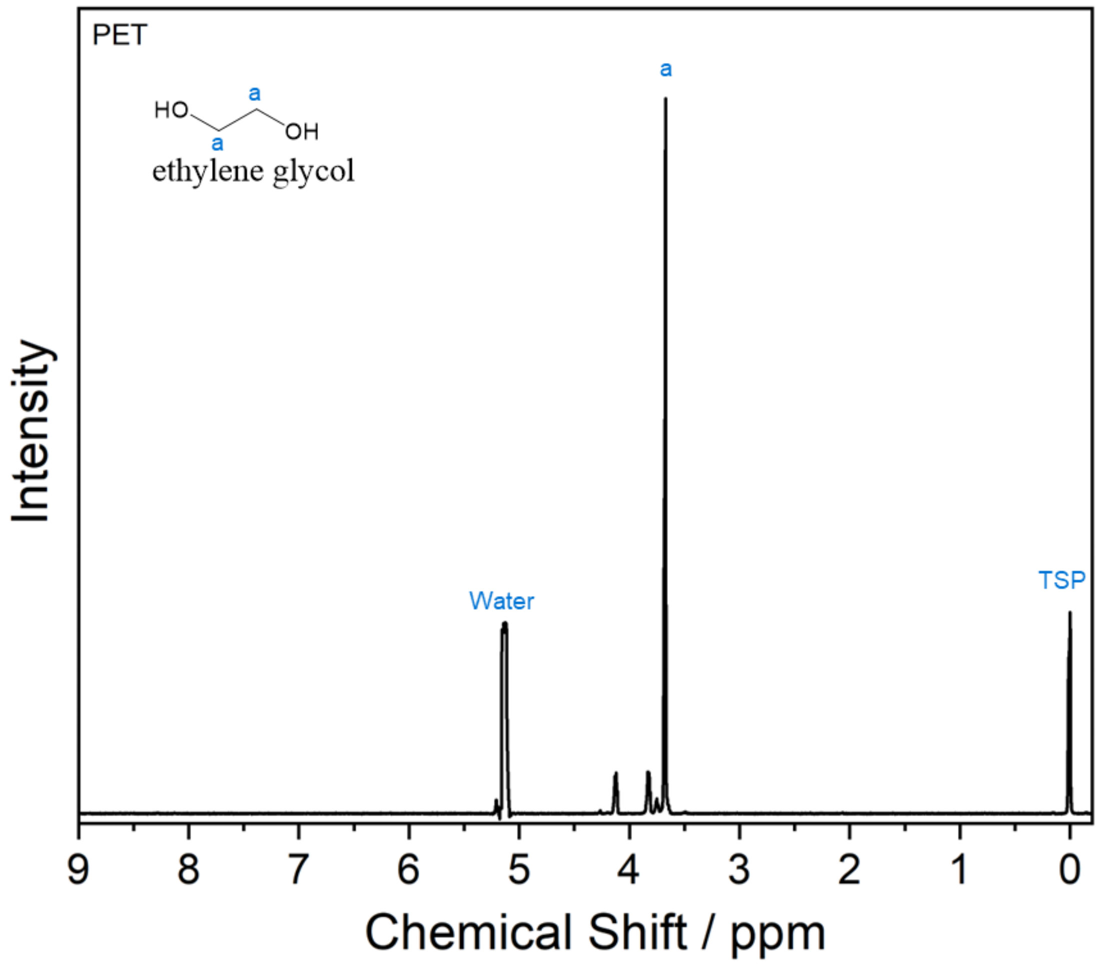

Fig. S10 | ^{1}H NMR spectra of acid hydrolysate from PET depolymerization (7.5 M H_2SO_4 at 140 °C for 6 hours). Peak at 0 is TSP in D_2O as NMR reference.

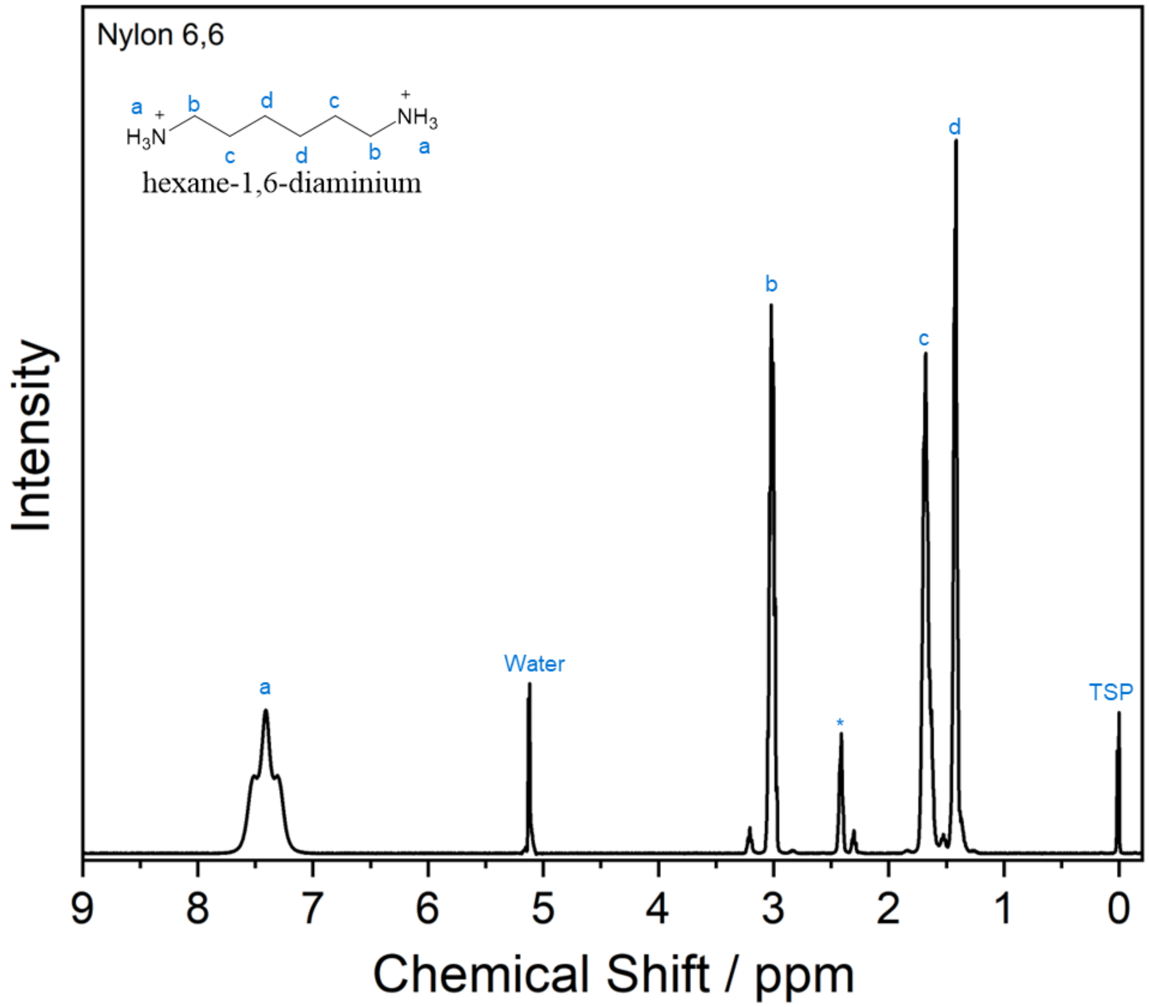

Fig. S11 | ^{1}H NMR spectra of acid hydrolysate from Nylon 66 depolymerization (7.5 M H_2SO_4 at 140 °C for 6 hours). Peak at 0 is TSP in D_2O as NMR reference. * refers to peaks corresponding to adipic acid.

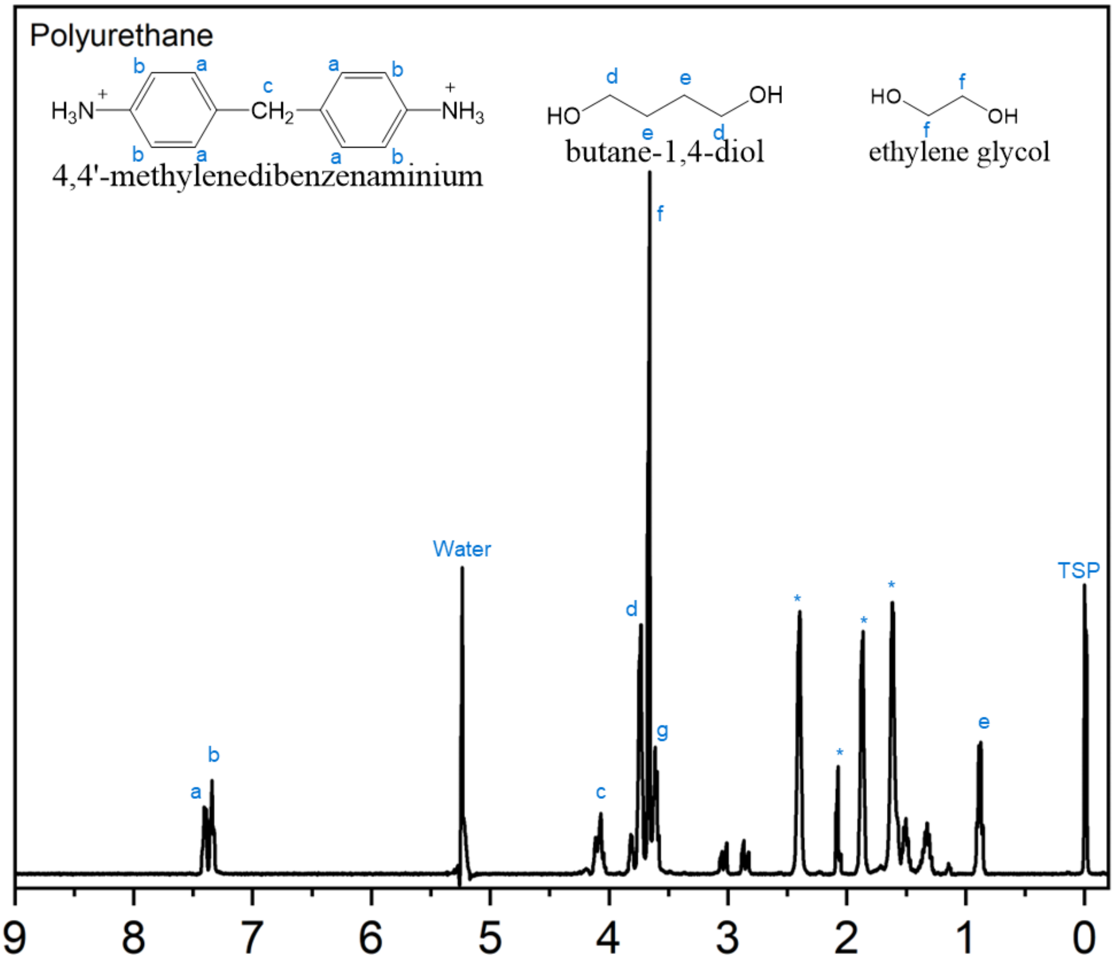

Fig. S12 | ^{1}H NMR spectra of acid hydrolysate from polyurethane depolymerization. Peak at 0 is TSP in D_2O as NMR reference. * refers to unidentified peaks suspected to be additive.[4]

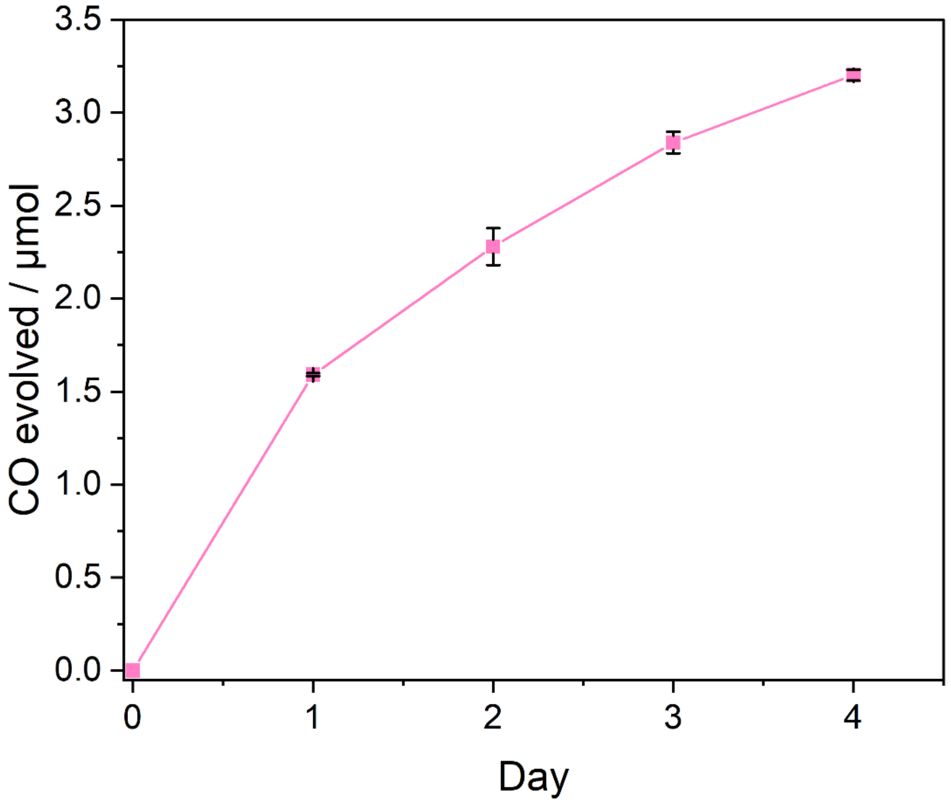

Fig. S13 | CO evolution experiments in 4 days from photoreforming of EG derived from PET through acid hydrolysis.[4] Acid hydrolysate was diluted 4-fold and added to acetonitrile of same volume. Reaction was conducted at room temperature and pressure under 405 nm LED irradiation.

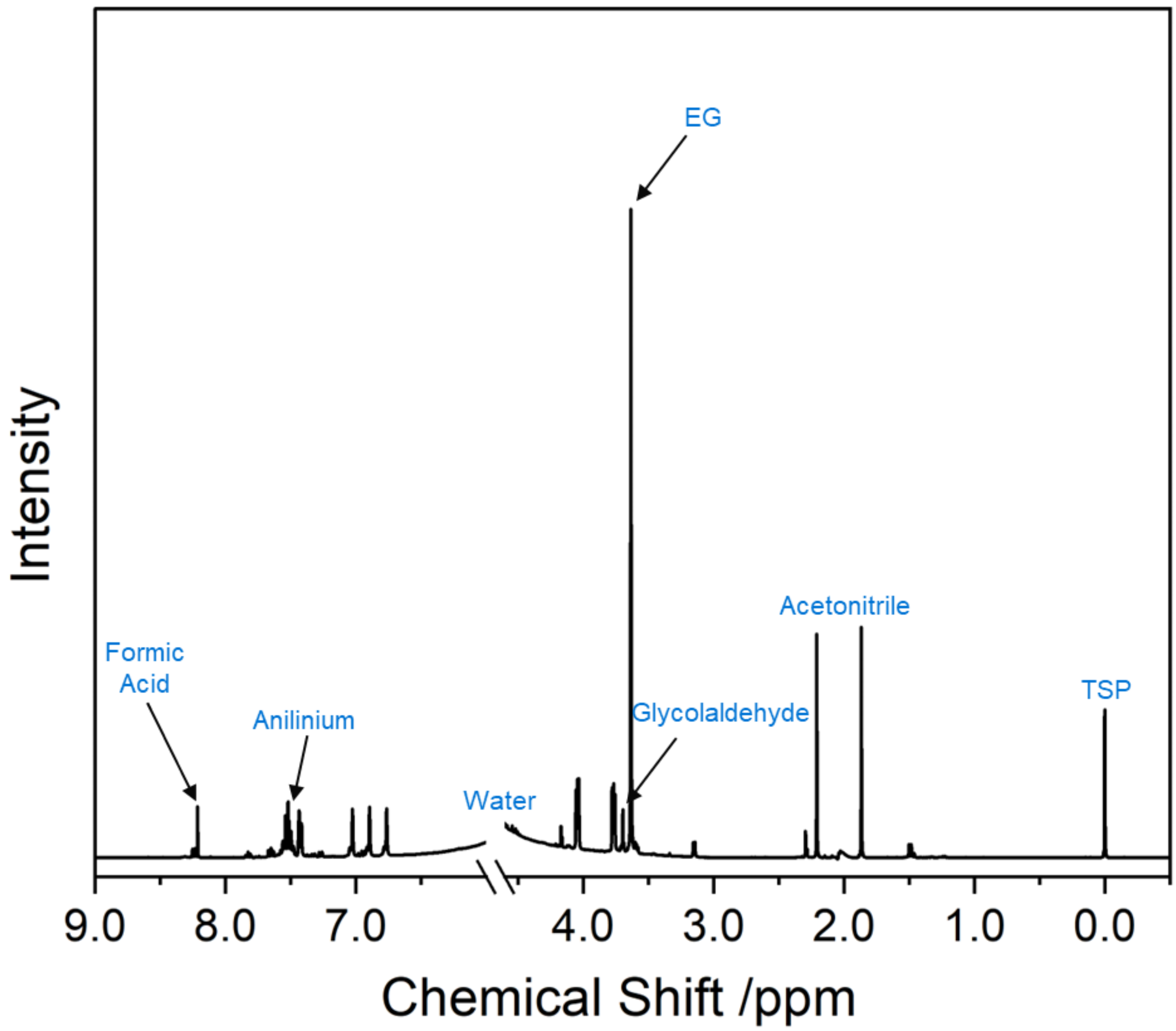

Fig. S14 | ^{1}H NMR spectra of reaction solution after photocatalytic transfer hydrogenation of nitrobenzene using acid hydrolysate from PET depolymerization. Peak at 0 is TSP in D_2O as NMR reference. Photocatalytic transfer hydrogenation was conducted under 405 nm irradiation at 25 °C for 24 hours.

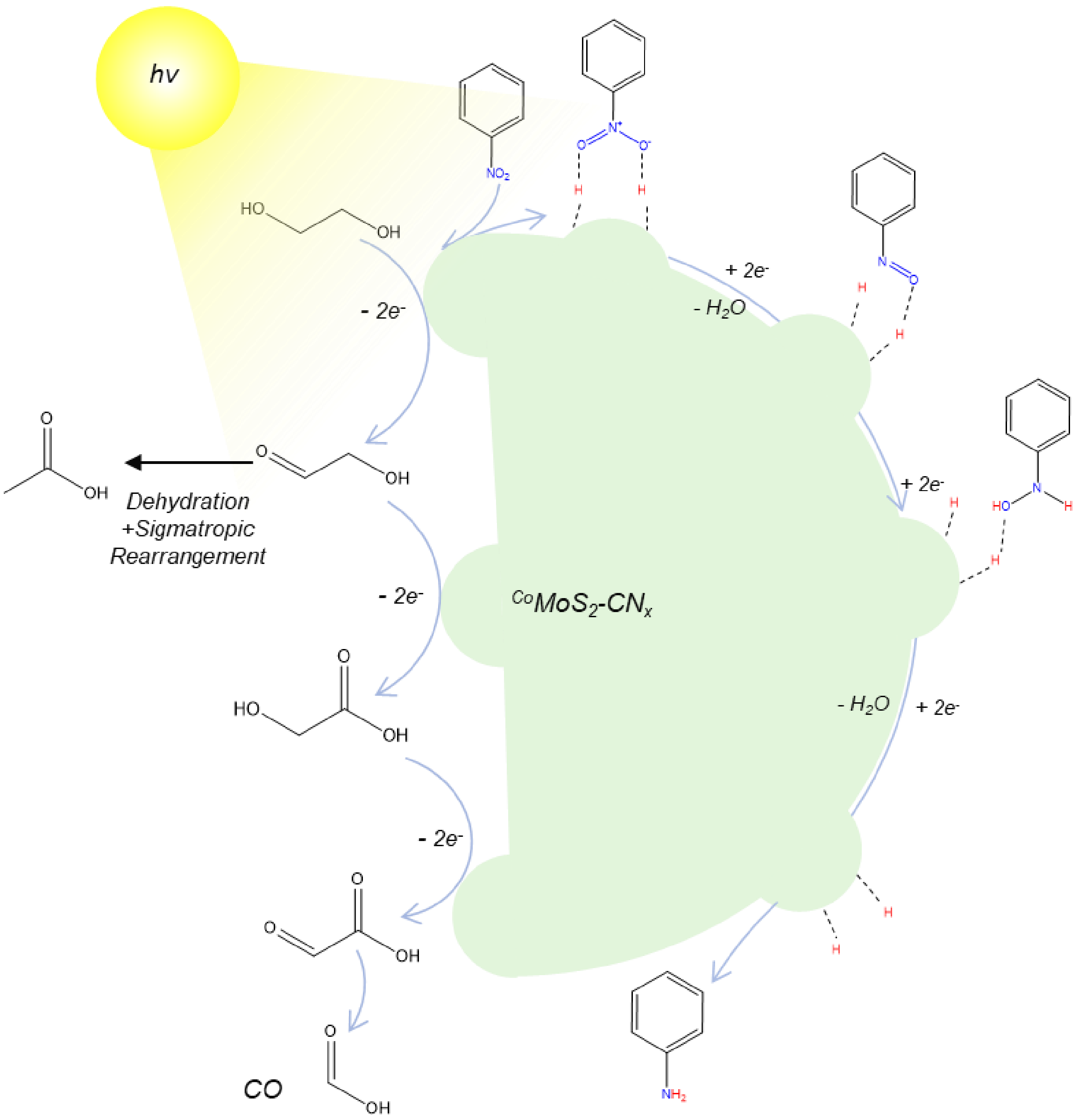

Fig. S15 | Proposed photocatalytic transfer hydrogenation reaction mechanism with nitrobenzene as substrate and aniline as product. Mechanism shows 6-electron reduction of with glycolaldehyde and formic acid as liquid products and CO as main gaseous product from minor pathway. Acid hydrolysate was diluted 4-fold and added to acetonitrile of same volume. Reaction was conducted at room temperature and pressure under 405 nm LED irradiation.

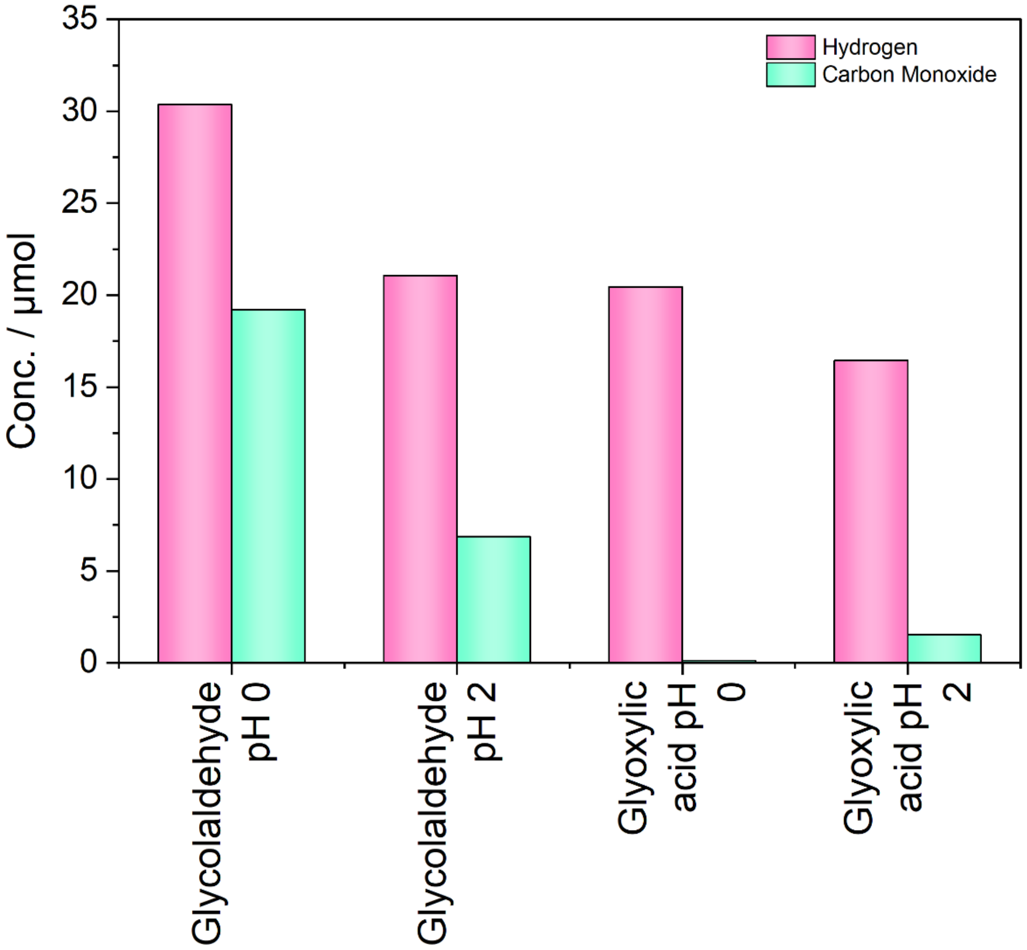

Fig. S16 | Comparison of H_2 and CO evolved in 4 hours from photoreforming of glycolaldehyde and glyoxylic acid at pH 2 and pH 0. Solvent system is 1:1 H_2O:MeCN. Reaction was conducted at room temperature and pressure under 405 nm LED irradiation and confirms CO is an oxidation product and not degradation product of carbon nitride.

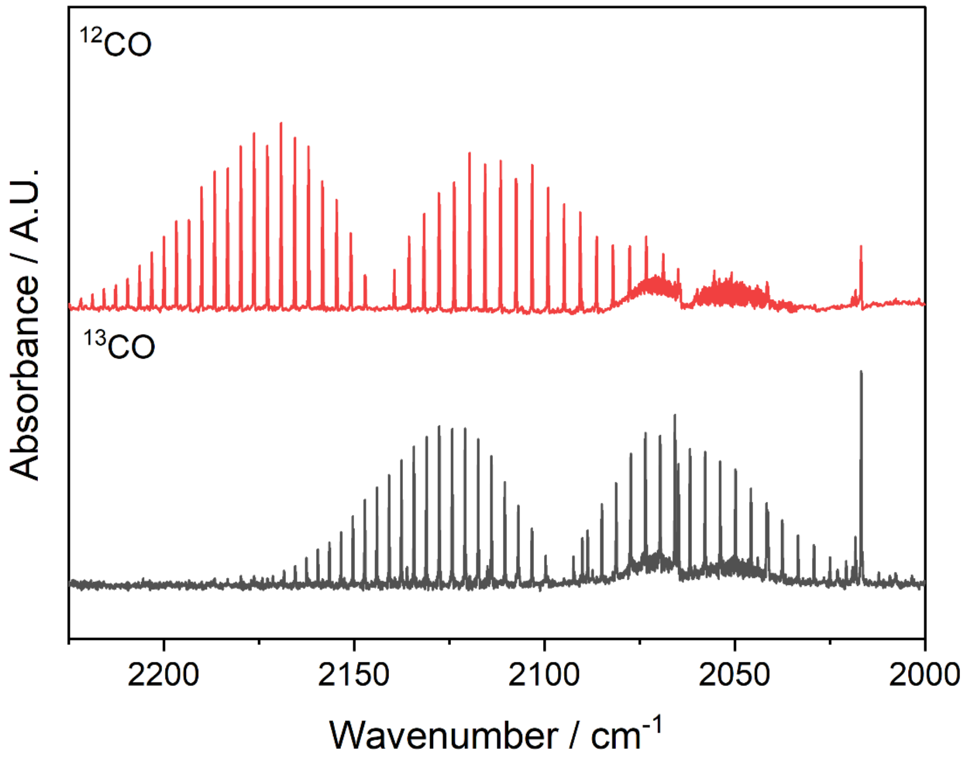

Fig. S17 | Gas phase IR of gaseous product from ^{12}C and ^{13}C ethylene glycol oxidation under acidic conditions. 40 mM of ethylene glycol with 20 mg of $^{Co}MoS_2$-CN_x in 1 mL solution was irradiated using 405nm LEDs for 24 hours.

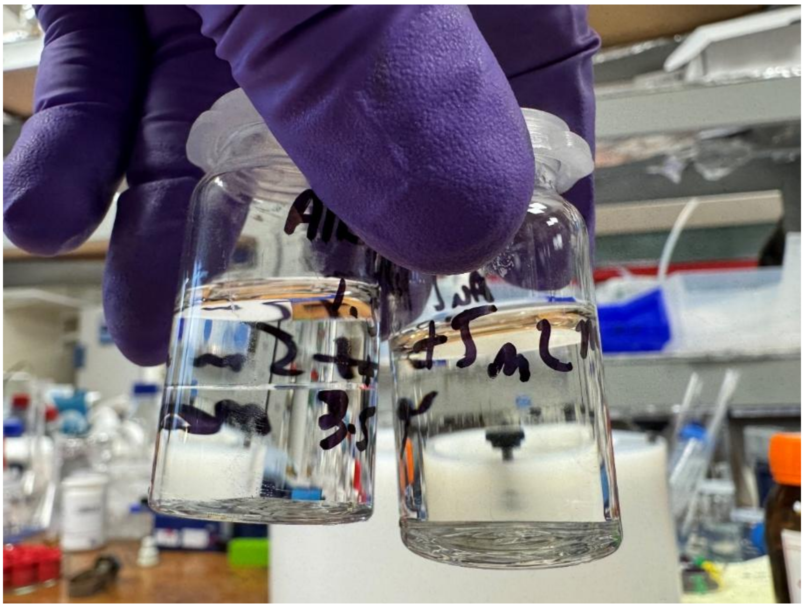

Fig. S18 | Image showing phase separation between alkaline hydrolysate mimic and acetonitrile on the left and complete miscibility between acid hydrolysate and acetonitrile.

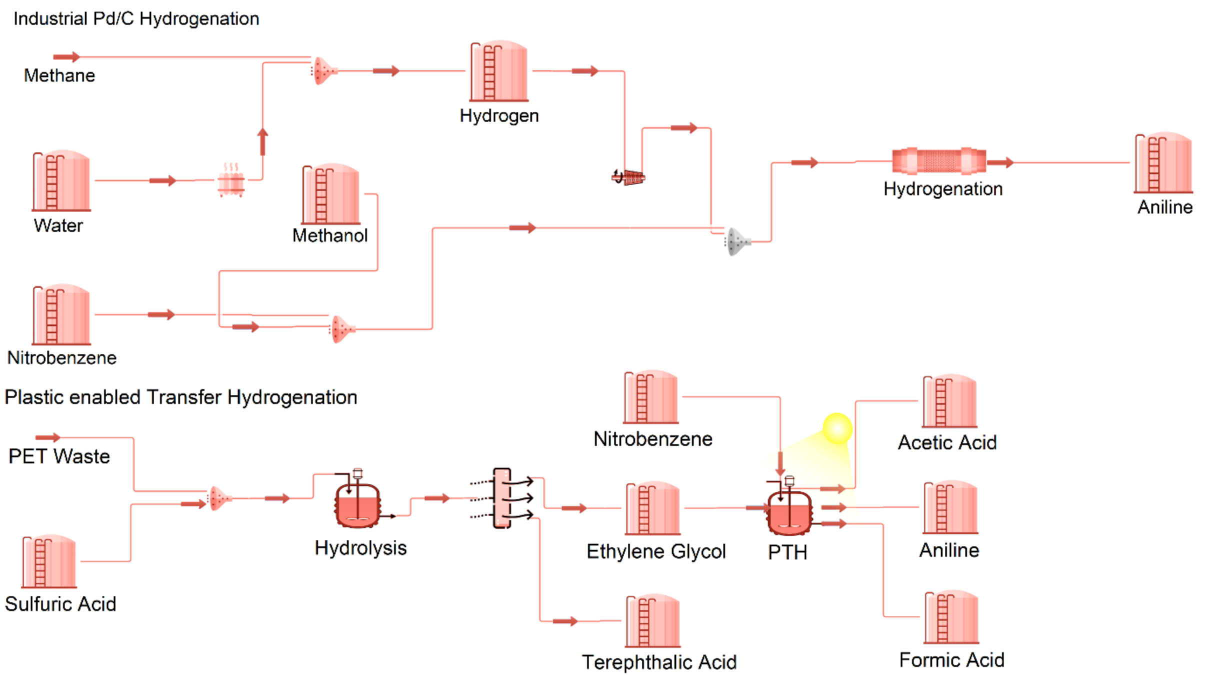

Fig. S19 | Technoeconomic and environmental comparison of conventional and photocatalytic transfer hydrogenation routes to aniline. A. Process flow diagrams for conventional Pd/C hydrogenation using hydrogen from steam methane reforming (SMR) – top and plastic-assisted photocatalytic transfer hydrogenation (PTH) using PET-derived EG - bottom.

Supporting references

[1] G. Brieger, T. J. Nestrick, *Chem. Rev.* 1974, *74*, 567–580.
[2] D. Wang, D. Astruc, *Chem. Rev.* 2015, *115*, 6621–6686.
[3] Z.-W. Xi, L. Yang, D.-Y. Wang, C.-D. Pu, Y.-M. Shen, C.-D. Wu, X.-G. Peng, *J. Org. Chem.* 2018, *83*, 11886–11895.
[4] P. K. Kwarteng, Y. Liu, C. Han, S. A. Bonke, C. Pulignani, E. Reisner, *Joule* 2025, accepted.
[5] R. M. Mironenko, et al. *ChemCatChem* 2021, *13*, 3656–3661.
[6] S. Duraisamy, et al. *ACS Appl. Nano Mater.* 2021, *4*, 2642–2656.
[7] P. Lara, K. Philippot, *Catal. Sci. Technol.* 2014, *4*, 2445–2465.
[8] S. K. Tanielyan, et al. *Org. Process Res. Dev.* 2007, *11*, 681–688.
[9] M. Turáková, et al. *Appl. Catal. A* 2015, *499*, 66–76.
[10] F. Cárdenas-Lizana, et al. *ACS Catal.* 2013, *3*, 1386–1396.
[11] F. Gallucci, A. Comite, G. Capannelli, A. Basile, *Ind. Eng. Chem. Res.* 2006, *45*, 2994–3000.
[12] International Energy Agency, *Global Hydrogen Review 2023*, IEA, Paris, 2023.
[13] S. Bugosen, I. D. Mantilla, F. Tarazona-Vasquez, *Heliyon* 2020, *6*, e05778.
[14] UK Chemical Suppliers, "Ethylene glycol UK suppliers, bulk distributors, industrial manufacturers and wholesalers", https://www.ukchemicalsuppliers.co.uk/content/page/ethylene-glycol (accessed: May 8, 2025).
[15] T. Uekert, C. M. Pichler, T. Schubert, E. Reisner, *Nat. Sustain.* 2021, *4*, 383–391.
[16] H. M. U. Ayub, et al. *Process Saf. Environ. Prot.* 2024, *186*, 921–932.
[17] Chemanalyst, "Melamine price: market analysis and forecast", https://www.chemanalyst.com/industry-report/melamine-market-812 (accessed: May 8, 2025).
[18] Metal.com, "Cobalt oxide price today: historical price charts", https://www.metal.com/en/prices/201102250301 (accessed: May 8, 2025).
[19] Chemanalyst, "Sodium thiocyanate prices and trend", https://www.chemanalyst.com/Pricing-data/sodium-thiocyanate-1547 (accessed: May 8, 2025).
[20] Chemanalyst, "Sulphuric acid price", https://www.chemanalyst.com/Pricing-data/sulphuric-acid-70 (accessed: May 8, 2025).
[21] Lab-Shop, "Acetonitrile (general reagent grade) price listing", https://www.lab-shop.com/chemicals-reagents-c84/fisher-chemical-acetonitrile-general-reagent-grade-99-0-specified-laboratory-reagent-slr-extra-pure-200l-a-0620-27-p913 (accessed: May 8, 2025).
[22] Methanex, "Methanol pricing", https://www.methanex.com/our-products/about-methanol/pricing/ (accessed: May 8, 2025).
[23] Chemanalyst, "Formaldehyde pricing", https://www.chemanalyst.com/Pricing-data/formaldehyde-1214 (accessed: May 8, 2025).
[24] J. Saleem, et al. *Sci. Rep.* 2025, *15*, 32797.
[25] GlobeNewswire Online, "Palladium chloride production analysis and price update 2025", https://globenewswire.online/news-release-global-palladium-chloride-production-analysis-import-export-price-update-2025/ (accessed: May 8, 2025).
[26] Intralabs, "Sodium bicarbonate price listing", https://www.intralabs.co.uk/sodium-bicarbonate.html (accessed: May 8, 2025).
[27] G. Towler, R. Sinnott, *Chemical Engineering Design: Principles, Practice and Economics of Plant and Process Design*, Butterworth-Heinemann, Oxford, 2021.
[28] K. D. Timmerhaus, R. E. West, *Plant Design and Economics for Chemical Engineers*, McGraw-Hill, New York, 2004.
[29] W. D. Seider, et al. *Product and Process Design Principles: Synthesis, Analysis and Evaluation*, Wiley, Hoboken, 2016.
[30] S. Bhattacharjee, S. Linley, E. Reisner, *Nat. Rev. Chem.* 2024, *8*, 87–105.
[31] NATPOW, "Product listing (power supply)", https://www.amazon.co.uk/stores/NATPOW/page/2008E265-265A-

4AF3-8B6B-9DD7E5E3D8EE (accessed: May 8, 2025).
[32] M. R. Shaner, H. A. Atwater, N. S. Lewis, E. W. McFarland, *Energy Environ. Sci.* 2016, *9*, 2354–2371.
[33] M. Lauer, "Economics methodology guideline", in *Workshop WP3B*, Joanneum Research Graz, Graz, 2023.
[34] S. Bhattacharjee, et al. *J. Am. Chem. Soc.* 2023, *145*, 20355–20364.
[35] M. F. Ashby, *Materials and the Environment: Eco-informed Material Choice*, Elsevier, Oxford, 2012.
[36] G. Poszmik, H. Keller, J. Choo, "Estimating the impact of using recycled PTFE on CO_2 emissions", https://shamrocktechnologies.com/co2-emissions/ (accessed: May 8, 2025).
[37] CarbonCloud, "Climate footprint of plastic, HDPE resin, fossil based: technical report", https://apps.carboncloud.com/climatehub/product-reports/id/128796899116 (accessed: May 8, 2025).
[38] A. Alsabri, S. G. Al-Ghamdi, *Energy Rep.* 2020, *6*, 364–370.
[39] K. Shahzad, et al. *Chem. Eng.* 2015, *45*, 1–6.
[40] Carbon Brief, "UK's electricity was cleanest ever in 2024", https://www.carbonbrief.org/analysis-uks-electricity-was-cleanest-ever-in-2024/ (accessed: May 8, 2025).
[41] Climatiq, "Emission factor: melamine", https://www.climatiq.io/data/emission-factor/55b31136-d149-4fb1-a15d-121e1cb94293 (accessed: May 8, 2025).
[42] International Molybdenum Association, "Executive summary", in *The Voice of the Molybdenum Industry*, IMOA, 2024.
[43] Cobalt Institute, "Life cycle assessment of cobalt and cobalt compounds", https://www.cobaltinstitute.org/responsible-sustainable-cobalt/life-cycle-assessment/ (accessed: May 8, 2025).
[44] J. Adeniran, R. Yusuf, M. Adetoro, *J. Eng. Technol.* 2017, *8*, 1–25.
[45] A. Tripodi, et al. *ACS Sustain. Chem. Eng.* 2018, *6*, 5441–5451.
[46] ecoinvent Association, "Nitrobenzene production, UPR, ecoinvent 3.6 consequential dataset", https://www.globallcadataaccess.org/nitrobenzene-production-upr-ecoinvent-36-consequential (accessed: May 8, 2025).
[47] S. U. Odabasi, H. Buyukgungor, in *1st Int. Black Sea Congr. Environ. Sci. (IBCESS)*, Turkey, Aug 31–Sep 3, 2017.
[48] Worldstainless, "CO_2 emissions report 2024", https://worldstainless.org/pdf-viewer/viewer.html?file=https%3A%2F%2Fworldstainless.org%2Fwp-content%2Fuploads%2F2025%2F02%2Fworldstainless_CO2_Emissions_Report.pdf (accessed: May 8, 2025).
[49] Climatiq, "Emission factor: palladium", https://www.climatiq.io/data/emission-factor/10073cff-f35a-47c0-a401-b21aa178f0dc (accessed: May 8, 2025).
[50] Euro Chlor, "Eco-profile and environmental product declaration of the European chlor-alkali industry", https://www.eurochlor.org/ (accessed: May 8, 2025).
[51] CarbonCloud, "Carbon footprint of sodium carbonate (Na_2CO_3), E500", https://apps.carboncloud.com/climatehub/product-reports/id/44158023257 (accessed: May 8, 2025).
[52] CarbonCloud, "Carbon footprint of salt (NaCl)", https://apps.carboncloud.com/climatehub/product-reports/id/5403842093 (accessed: May 8, 2025).
[53] J. Puhar, D. Krajnc, L. Čuček, A. Vujanović, *J. Clean. Prod.* 2022, *377*, 134537.
[54] A. Vardaro, F. Arfelli, F. Passarini, D. Cespi, *Integr. Environ. Assess. Manag.* 2025, vjaf129.
[55] H. H. Cho, V. Strezov, T. J. Evans, *Energy Rep.* 2022, *8*, 13585–13595.
[56] M. A. Khan, C. Young, C. Mackinnon, D. B. Layzell, *Transition Accelerator Tech. Briefs* 2021, *1*, 1–36.
